# Do Non-Invasive Fibrosis Indices Reflect Interstitial Lung Disease and Pulmonary Function in Systemic Sclerosis?

**DOI:** 10.3390/diagnostics16213575

**Published:** 2026-10-29

**Authors:** Ertugrul Cagri Bolek, Ibrahim Karaduman, Ibrahim Vasi, Hatice Avsar, Melek Beril Kenarli, Arzu Bicer, Safa Senel, Esra Kizmaz Gunesli, Busra Koksoy, Burak Karakaya, Esma Eseroglu, Ibrahim Yahya Cakir, Nilgun Yilmaz Demirci, Hamit Kucuk, Abdulsamet Erden

**Affiliations:** 1 Division of Rheumatology, Department of Internal Medicine, Faculty of Medicine, Gazi University, 06560 Ankara, Turkey; 2 Department of Internal Medicine, Faculty of Medicine, Gazi University, 06560 Ankara, Turkey; 3 Department of Pulmonology, Faculty of Medicine, Gazi University, 06560 Ankara, Turkey

**Keywords:** systemic sclerosis, interstitial lung disease, FIB-4 index, APRI, pulmonary function tests, forced vital capacity, fibrosis, biomarkers

## Abstract

**Background/Objectives**: Interstitial lung disease (ILD) is a major manifestation of systemic sclerosis (SSc). The Fibrosis-4 index (FIB-4) and aspartate aminotransferase-to-platelet ratio index (APRI) are indirect hepatic fibrosis indices of uncertain pulmonary relevance. We compared these indices by documented ILD status and explored associations with pulmonary function and historical forced vital capacity (FVC) changes. **Methods**: This retrospective observational study included 102 patients with SSc (61 with ILD, 41 without ILD). Indices were calculated from the last eligible laboratory assessment; ILD was classified using available high-resolution computed tomography records. Patients with relevant hepatic and laboratory confounders were excluded. Pulmonary associations were explored within SSc-ILD, including a sensitivity analysis restricted to ≤12 months between laboratory and follow-up pulmonary function testing. **Results**: Neither index differed significantly by ILD status (FIB-4, *p* = 0.91; APRI, *p* = 0.06). In logistic models adjusted for age and diffuse cutaneous phenotype, FIB-4 showed a borderline inverse association with documented ILD status only after adjustment for age and and diffuse cutaneous phenotype (adjusted odds ratio 0.247, 95% confidence interval 0.062–0.988; *p* = 0.048), whereas APRI did not (*p* = 0.064). Within SSc-ILD, neither index correlated significantly with FVC % predicted, diffusing capacity for carbon monoxide (DLCO) % predicted, forced expiratory volume in 1 s (FEV1) % predicted, or historical FVC changes. FIB-4 correlated inversely with absolute FVC (ρ = −0.53, *p* < 0.001), but this association was not significant after age and sex adjustment (*p* = 0.40). The absolute-FVC correlation persisted within the ≤12-month subgroup. **Conclusions**: Findings do not consistently support these indices as stand-alone indicators of SSc pulmonary involvement; prospective validation is needed.

## 1. Introduction

Systemic sclerosis (SSc) is a complex autoimmune connective tissue disease characterized by immune dysregulation, vasculopathy, and progressive fibrosis of the skin and internal organs [1]. Among its visceral manifestations, interstitial lung disease (ILD) is particularly important because of its high prevalence and substantial contribution to SSc-related morbidity and mortality [2,3]. High-resolution computed tomography (HRCT) remains the principal imaging modality for detecting SSc-associated ILD (SSc-ILD). In contrast, serial pulmonary function tests (PFTs), particularly forced vital capacity (FVC) and diffusing capacity of the lung for carbon monoxide (DLCO), are central to evaluating functional impairment and monitoring disease course [2,3]. Nevertheless, SSc-ILD is clinically heterogeneous: some patients remain physiologically stable for prolonged periods, whereas others develop progressive functional decline. This heterogeneity has stimulated interest in accessible biomarkers that could complement established radiological and physiological assessments.

Fibrosis in SSc is not restricted to a single organ; therefore, circulating markers reflecting extracellular matrix turnover have been investigated as potential indicators of systemic fibrotic burden. An important example is the enhanced liver fibrosis (ELF) test, initially developed and validated to assess hepatic fibrosis but subsequently evaluated in SSc. In an early SSc study, the ELF score was associated with the extent of skin involvement, DLCO, overall disease severity, and disease activity [4]. These findings were subsequently supported in a European multicenter cohort, in which ELF and its individual components were associated with skin involvement and several pulmonary functional measures, including FVC, total lung capacity (TLC), and DLCO [5]. These observations support the broader concept that serum-based fibrosis-related indices originally developed in another organ system may contain information relevant to the systemic fibrotic phenotype of SSc; however, such associations do not establish organ specificity or imply that these indices directly quantify pulmonary fibrosis.

The Fibrosis-4 index (FIB-4) and the aspartate aminotransferase-to-platelet ratio index (APRI) are simpler serum-based indices calculated from routinely available clinical and laboratory variables. FIB-4 incorporates age, aspartate aminotransferase (AST), alanine aminotransferase (ALT), and platelet count and was initially developed to identify hepatic fibrosis in patients with human immunodeficiency virus/hepatitis C virus coinfection [6]. In contrast, APRI combines AST relative to its upper limit of normal with platelet count and was originally developed in chronic hepatitis C [7]. Both indices are inexpensive and require no specialized assays or imaging. Although clinically overt hepatic involvement is considered relatively uncommon in SSc, studies using transient elastography have demonstrated that increased liver stiffness may occur even in patients without previously recognized liver disease [8,9]. These observations also emphasize the importance of distinguishing potential systemic associations of FIB-4 and APRI from elevations caused by primary hepatic disease or other conditions affecting aminotransferases or platelet counts.

Interest in the possible extrahepatic significance of these indices has recently extended to fibrotic lung diseases. In idiopathic pulmonary fibrosis (IPF), Cocconcelli et al. reported subclinical liver fibrosis in more than one-third of their cohort and found APRI to be independently associated with survival [10]. More recently, Koyun et al. reported that higher FIB-4 values were associated with mortality in patients with IPF [11]. However, the available evidence is not uniform.

In a study comparing idiopathic and secondary pulmonary fibrosis, FIB-4 was higher in patients with IPF [12]. In contrast, APRI showed less discriminatory value, and pulmonary function did not differ significantly across hepatic fibrosis risk categories [12]. Thus, the biological interpretation of these associations remains uncertain. Elevated FIB-4 or APRI in fibrotic lung disease could theoretically reflect shared profibrotic or systemic inflammatory pathways, occult hepatic involvement, or nonspecific effects of aging, aminotransferase abnormalities, and platelet variation rather than pulmonary fibrosis itself. Accordingly, FIB-4 and APRI should currently be regarded as liver-derived non-invasive fibrosis indices with potential extrahepatic associations, rather than as validated direct biomarkers of lung fibrosis.

Although FIB-4 and APRI have previously been evaluated in SSc populations, primarily in the context of hepatic involvement [8], their potential relevance to the broader spectrum of extrahepatic fibrotic manifestations in SSc remains insufficiently characterized. Previous investigations of fibrosis-related biomarkers in SSc have addressed hepatic stiffness and extracellular matrix-based fibrosis panels rather than the extrahepatic applicability of routinely calculable indices such as FIB-4 and APRI [4,5,8,9]. In particular, their associations with documented SSc-ILD status, pulmonary function, and historical FVC changes remain insufficiently investigated. This study primarily aimed to compare FIB-4 and APRI between SSc patients with and without documented ILD, while secondarily assessing, in an exploratory manner, their associations with pulmonary function and historical first-to-follow-up FVC changes, particularly the annualized change in FVC percentage predicted, within the SSc-ILD subgroup.

## 2. Materials and Methods

### 2.1. Study Design and Population

This was a single-center, retrospective observational study comprising a primary cross-sectional comparison of non-invasive fibrosis indices according to retrospectively documented SSc-ILD status and secondary exploratory analyses of pulmonary function, including historical longitudinal FVC changes. Patients were identified based on the study eligibility criteria and subsequently classified according to documented ILD status. The study was not designed to investigate FIB-4 or APRI as baseline predictors of incident ILD or subsequent pulmonary function decline.

No a priori sample-size calculation was performed because this was a retrospective observational study based on the available institutional records. The analytical sample comprised all identified patients who fulfilled the prespecified eligibility criteria during the study period. Thus, the final sample size was determined by patient eligibility and data availability rather than a prospectively defined statistical power target.

The study was conducted at the Division of Rheumatology, Department of Internal Medicine, Gazi University Faculty of Medicine, a tertiary referral center. Institutional records were screened to identify patients diagnosed with SSc between 1 January 2016 and 1 January 2026. Patients fulfilling the 2013 American College of Rheumatology/European League Against Rheumatism (ACR/EULAR) classification criteria for SSc were eligible for inclusion [13]. Our group previously characterized the institutional SSc-ILD population and some clinical outcomes [14,15].

Patients were classified according to documented SSc-associated ILD status in the available clinical records, with ILD identified on the basis of HRCT findings. The exact dates of initial ILD documentation were not available for analysis in all records; therefore, the temporal relationship between ILD documentation and the laboratory assessment used to calculate FIB-4 and APRI could not be established for individual patients.

To minimize potential confounding of FIB-4 and APRI by hepatic and other non-pulmonary conditions, patients with known chronic liver disease were excluded. Hepatic exclusion criteria included chronic hepatitis B or C infection, autoimmune hepatitis, primary biliary cholangitis, primary sclerosing cholangitis, and other documented chronic hepatobiliary disease. Patients with clinically documented active overlap myositis were also excluded because muscle injury may elevate aminotransferase levels independently of hepatic disease. Myositis-related exclusions were based on available clinical documentation. Creatine kinase (CK) measurements were not systematically available for all patients and therefore were not used as a standardized exclusion criterion.

For the last eligible laboratory assessment, we also reviewed medical records for acute conditions that could substantially alter hematological or biochemical parameters. Laboratory measurements obtained during clinically significant acute infection, active malignancy or cancer-related systemic illness, suspected drug-induced hepatotoxicity, or another acute condition considered likely to affect AST, ALT, or platelet count materially were not used for the primary fibrosis-index analysis. Patients without an eligible laboratory assessment allowing calculation of both FIB-4 and APRI were excluded from the final analytical cohort.

### 2.2. Demographic, Clinical, and Serological Assessment

Demographic and clinical information was extracted retrospectively from electronic medical records and the institutional SSc database. Recorded variables included sex, age at SSc diagnosis, age at the last available clinical assessment, disease duration, smoking history, SSc cutaneous subtype, major clinical manifestations, serological characteristics, treatment exposure during follow-up, and vital status where available. Age at the last eligible laboratory assessment was used for FIB-4 calculation and as the age covariate in the adjusted regression models.

Patients were classified as having limited cutaneous systemic sclerosis (lcSSc) or diffuse cutaneous systemic sclerosis (dcSSc) according to the LeRoy classification [16]. In lcSSc, skin sclerosis was restricted to areas distal to the elbows and knees, with or without facial involvement. In contrast, dcSSc was characterized by skin involvement extending proximal to the elbows and/or knees and/or involving the trunk [16]. Patients fulfilling SSc classification criteria without clinically detectable skin sclerosis were categorized as systemic sclerosis sine scleroderma. For regression analyses, dcSSc was compared with a non-diffuse category comprising lcSSc and SSc sine scleroderma; patients with missing cutaneous subtype data were excluded from models requiring this variable.

Serological variables included antinuclear antibody (ANA) positivity and SSc-associated autoantibodies, particularly anti-centromere and anti-topoisomerase I antibodies. Clinical manifestations assessed during follow-up included Raynaud phenomenon, history of digital ulcers, telangiectasia, gastrointestinal involvement, and documented pulmonary hypertension.

Clinical manifestations and treatment exposures were retrospectively extracted from medical records throughout the documented disease course and generally reflected ever-documented characteristics. These variables did not necessarily represent manifestations present or treatments ongoing at the time of the last eligible laboratory assessment.

### 2.3. Assessment of Interstitial Lung Disease

ILD status was retrospectively determined from the available HRCT findings and clinical documentation. Available HRCT examinations were assessed by a pulmonologist with more than 20 years of clinical experience in interstitial lung diseases (N.Y.D.). No additional independent image review was performed, and the assessment was not blinded to clinical or pulmonary function data. Consequently, interobserver agreement and formal reader adjudication were not assessed. Borderline or equivocal HRCT findings were discussed in a multidisciplinary rheumatology–pulmonology meeting to inform ILD classification.

HRCT was not uniformly available in patients classified as having no documented ILD, and the exact proportion with available HRCT could not be established from the analytical dataset. As this was a retrospective chart review, HRCT examinations were not performed according to a standardized screening protocol. Therefore, the comparison group was defined by the absence of documented ILD rather than systematic HRCT confirmation of the absence of ILD.

Patients were categorized as having SSc-ILD when HRCT demonstrated findings compatible with interstitial involvement, including reticular abnormalities, ground-glass opacities, traction bronchiectasis, and/or honeycombing in an appropriate clinical context. The assessment of SSc-ILD followed the approach previously used in our institutional cohorts [14,15].

### 2.4. Laboratory Assessment and Calculation of Non-Invasive Fibrosis Indices

For the primary comparison of patients with and without documented ILD, FIB-4 and APRI were calculated using AST, ALT, and platelet counts from each patient’s last eligible laboratory assessment, with all three measurements available from the same assessment. Age was calculated at the date of the corresponding laboratory assessment.

FIB-4 was calculated according to the originally described formula [6]:

$$\mathrm{FIB}\text{-}4\;=\;[\mathrm{Age}\;(\mathrm{years})\;\times \;\mathrm{AST}\;(U/L)]/[\mathrm{platelet}\;\mathrm{count}\;(10^{9}/L)\;\times \;\surd \mathrm{ALT}\;(U/L)]$$

APRI was calculated according to the original formula described by Wai et al. [7]:

$$\mathrm{APRI}\;=\;[(\mathrm{AST}\;(U/L)/\mathrm{AST}\;\mathrm{upper}\;\mathrm{limit}\;\mathrm{of}\;\mathrm{normal})/\mathrm{platelet}\;\mathrm{count}\;(10^{9}/L)]\;\times \;100$$

The AST upper limit of normal (ULN) applicable to the corresponding laboratory measurement was used for APRI calculation, thereby accounting for changes in laboratory-specific reference ranges over time. Platelet counts were expressed as 10^9^/L.

FIB-4 and APRI were primarily evaluated as continuous variables. Liver disease-specific diagnostic thresholds were not used to define the primary study groups because these thresholds were originally developed and validated for the assessment of hepatic fibrosis rather than pulmonary involvement in SSc [6,7].

For the primary comparative analysis, the date of the last eligible laboratory assessment used to calculate FIB-4 and APRI was designated as the laboratory index date. ILD status was determined retrospectively from the available clinical and HRCT records. This index date defined the timing of the fibrosis-index measurements and was not treated as the beginning of a prospective follow-up period for incident ILD or subsequent pulmonary function decline.

### 2.5. Pulmonary Function Assessment

PFTs were performed during routine clinical follow-up using the MasterScreen PFT Pro system (CareFusion, Höchberg, Germany). Spirometry was performed according to established American Thoracic Society/European Respiratory Society technical standards [17].

Pulmonary function parameters included forced vital capacity (FVC), expressed in liters and as a percentage of the predicted value; forced expiratory volume in one second (FEV1), expressed as a percentage of the predicted value; and the last available diffusing capacity of the lung for carbon monoxide (DLCO), expressed as a percentage of the predicted value, where available.

Within the SSc-ILD subgroup, cross-sectional analyses were based on the last available PFT recorded in the longitudinal dataset (hereafter, “follow-up PFT”). Patients with only a first PFT and no recorded follow-up PFT were not included in these cross-sectional analyses. FIB-4 and APRI were evaluated in relation to follow-up FVC and FEV1 measurements and the last available DLCO measurement, where available. DLCO was generally measured during the same pulmonary function assessment, although exact temporal alignment was not independently verified for all patients. The absolute interval between the last eligible laboratory assessment and the follow-up PFT was calculated for each patient with available measurements. Given the retrospective nature of the study and variability in assessment timing, the primary analysis included all available observations. A sensitivity analysis was performed in patients with an absolute laboratory–follow-up PFT interval of ≤12 months.

### 2.6. Longitudinal Pulmonary Function Assessment

For patients with SSc-ILD who had both first and follow-up PFT measurements, longitudinal changes were calculated between these two assessments. The follow-up PFT represented the last available PFT in the longitudinal dataset.

Change in FVC volume was calculated as:

ΔFVC (L)=Last FVC (L)−First FVC (L)

Absolute change in FVC percentage predicted, expressed in percentage points, was calculated as:

ΔFVC (%pred)=Last FVC (%pred)−First FVC (%pred)

Relative change in FVC was calculated as:

$$\mathrm{Relative}\;\mathrm{FVC}\;\mathrm{change}\;\left(\%\right)=\frac{\mathrm{Last}\;\mathrm{FVC}\;\left(\%\mathrm{pred}\right)-\mathrm{First}\;\mathrm{FVC}\;\left(\%\mathrm{pred}\right)}{\mathrm{First}\;\mathrm{FVC}\;\left(\%\mathrm{pred}\right)}\times 100$$

The principal annualized longitudinal measure was the annualized change in FVC percentage predicted, expressed in percentage points per year and calculated as:

$$\mathrm{Annualized}\;\mathrm{FVC}\;\mathrm{change}\left(\mathrm{percentage}\;\mathrm{points}/\mathrm{year}\right)=\frac{\mathrm{Last}\;\mathrm{FVC}\left(\%\mathrm{pred}\right)-\mathrm{First}\;\mathrm{FVC}\left(\%\mathrm{pred}\right)}{\mathrm{Interval}\;\mathrm{between}\;\mathrm{the}\;\mathrm{first}\;\mathrm{and}\;\mathrm{last}\;\mathrm{PFTs}\;(\mathrm{years})}$$

Accordingly, negative values indicated a decline in FVC over time, whereas positive values indicated improvement.

Annualized change in FVC volume was additionally calculated using FVC expressed in liters:

$$\mathrm{Annualized}\;\mathrm{FVC}\;\mathrm{change}\left(L/\mathrm{year}\right)=\frac{\mathrm{Last}\;\mathrm{FVC}\left(L\right)-\mathrm{First}\;\mathrm{FVC}\left(L\right)}{\mathrm{interval}\;\mathrm{between}\;\mathrm{the}\;\mathrm{first}\;\mathrm{and}\;\mathrm{last}\;\mathrm{PFTs}\;(\mathrm{years})}$$

Annualized relative FVC change was calculated by dividing the relative first-to-follow-up FVC change by the interval between the first and last PFTs in years:

$$\mathrm{Annualized}\;\mathrm{relative}\;\mathrm{FVC}\;\mathrm{change}\;(\%/\mathrm{year})\;=\;\frac{\mathrm{Relative}\;\mathrm{FVC}\;\mathrm{change}\;(\%)}{\mathrm{Interval}\;\mathrm{between}\;\mathrm{the}\;\mathrm{first}\;\mathrm{and}\;\mathrm{last}\;\mathrm{PFTs}\;(\mathrm{years})}\;$$

Because these variables were calculated from the difference between the first and last available measurements rather than from a repeated-measures model incorporating all intermediate observations, they were described as annualized changes rather than longitudinal slopes.

Associations between FIB-4 and APRI obtained at the last eligible laboratory assessment and recorded first-to-follow-up FVC changes were evaluated in an exploratory manner. These analyses were not intended to assess the prediction of subsequent pulmonary function decline.

Additional temporal sensitivity analyses examined associations between FIB-4 or APRI and follow-up FVC (in liters and percentage predicted) across nested subgroups defined by absolute laboratory–follow-up PFT intervals of ≤3, ≤6, and ≤12 months. Spearman correlation coefficients were recalculated within each subgroup, and the corresponding sample sizes were reported.

### 2.7. Statistical Analysis

Statistical analyses were performed using IBM SPSS Statistics version 24.0 (IBM Corp., Armonk, NY, USA). The distribution of continuous variables was evaluated using the Shapiro–Wilk test together with visual inspection of histograms and quantile–quantile (Q–Q) plots. Normally distributed continuous variables were summarized as mean ± standard deviation (SD), whereas non-normally distributed variables were presented as median and interquartile range (IQR). Categorical variables were expressed as numbers and percentages.

Comparisons between two independent groups were performed using the independent-samples Student’s *t* test for normally distributed continuous variables and the Mann–Whitney *U* test for non-normally distributed variables. Categorical variables were compared using the Pearson chi-square test or Fisher’s exact test, as appropriate. Associations between FIB-4 or APRI and pulmonary function parameters, including historical FVC changes, were assessed using Spearman’s rank correlation coefficient. Correlation analyses were visualized using heatmaps generated in Python (version 3.13.18; Google Colab environment) with the pandas (version 2.2.3), seaborn (version 0.13.2), and matplotlib (version 3.10.0) libraries. Heatmap cells displayed the correlation coefficient (ρ) with the corresponding *p*-value in parentheses, using a diverging color scale centered at zero to indicate the direction and magnitude of the associations. Supplementary Code S1 provides the Python code used to generate the heatmaps.

To account for potential confounding, exploratory univariable and multivariable binary logistic regression analyses were performed with documented SSc-ILD status as the dependent variable. FIB-4 and APRI were evaluated in separate multivariable models adjusted for age at the last eligible laboratory assessment and diffuse cutaneous phenotype. Covariates were selected based on clinical relevance and model parsimony rather than univariable statistical significance. Because only two patients had SSc sine scleroderma, cutaneous subtype was categorized as diffuse versus non-diffuse phenotype, with the latter comprising limited cutaneous SSc and sine scleroderma.

Given the limited size of the smaller outcome group, the multivariable models were restricted to the fibrosis index, age, and diffuse cutaneous phenotype to limit the risk of overfitting. Sex and disease duration were not included in the ILD logistic regression models; therefore, residual confounding could not be excluded.

The primary study comparison concerned FIB-4 and APRI values according to documented SSc-ILD status. Analyses involving pulmonary function parameters, historical FVC changes, and temporal sensitivity subgroups were considered secondary, exploratory, and hypothesis-generating. Given the retrospective design and the absence of a prespecified multiplicity-adjustment strategy, no correction for multiple comparisons was applied. Accordingly, all reported *p*-values were considered nominal, and the findings were interpreted as exploratory and hypothesis-generating rather than confirmatory.

Age was expressed per 10-year increase and APRI per 0.1-unit increase to facilitate interpretation of the odds ratios. Adjusted and unadjusted odds ratios (ORs) with 95% confidence intervals (CIs) were reported. Multicollinearity was assessed using variance inflation factors, and the linearity of continuous predictors in the logit was explored using Box–Tidwell terms. Model fit and potentially influential observations were assessed using the Hosmer–Lemeshow test and regression diagnostics. Given the limited sample size and the exploratory nature of the analyses, the models were not intended for risk prediction or causal inference.

Exploratory univariable and multivariable linear regression analyses were performed to assess associations between FIB-4 or APRI and follow-up FVC, expressed in liters and as percentage predicted, within the SSc-ILD subgroup. FIB-4 and APRI were evaluated in separate models for each FVC outcome. Multivariable models were adjusted for age at the last eligible laboratory assessment and sex. FIB-4 was analyzed per 1-unit increase and APRI per 0.1-unit increase. Unstandardized regression coefficients (B) with 95% confidence intervals were reported. These models evaluated associations with observed follow-up FVC values rather than subsequent pulmonary function decline.

Missing data were handled using an available-case approach, whereby each analysis included patients with non-missing values for the variables required for that analysis. No statistical imputation was performed. Accordingly, the number of observations varied across analyses. Longitudinal FVC changes were calculated only for patients with the relevant measurements at both the first and follow-up PFT assessments. The extent of missingness was reported using the number of available observations and the corresponding proportion of patients with missing data. Among patients with SSc-ILD, demographic and clinical characteristics and fibrosis indices were compared between patients with and without an available follow-up PFT to explore potential selection differences related to follow-up data availability.

All statistical tests were two-sided, and a *p*-value < 0.05 was considered nominally statistically significant.

### 2.8. Ethical Considerations

The study was conducted in accordance with the Declaration of Helsinki and was approved by the Ethics Committee of Gazi University Faculty of Medicine (Approval No. 2026-1347; 21 July 2026). A subsequent amendment to the study protocol was approved by the Gazi University Ethics Commission (Approval No. 2026-1827). Informed consent was not required because the study was retrospective and used de-identified clinical data.

## 3. Results

### 3.1. Study Population and Clinical Characteristics

**Figure 1:**
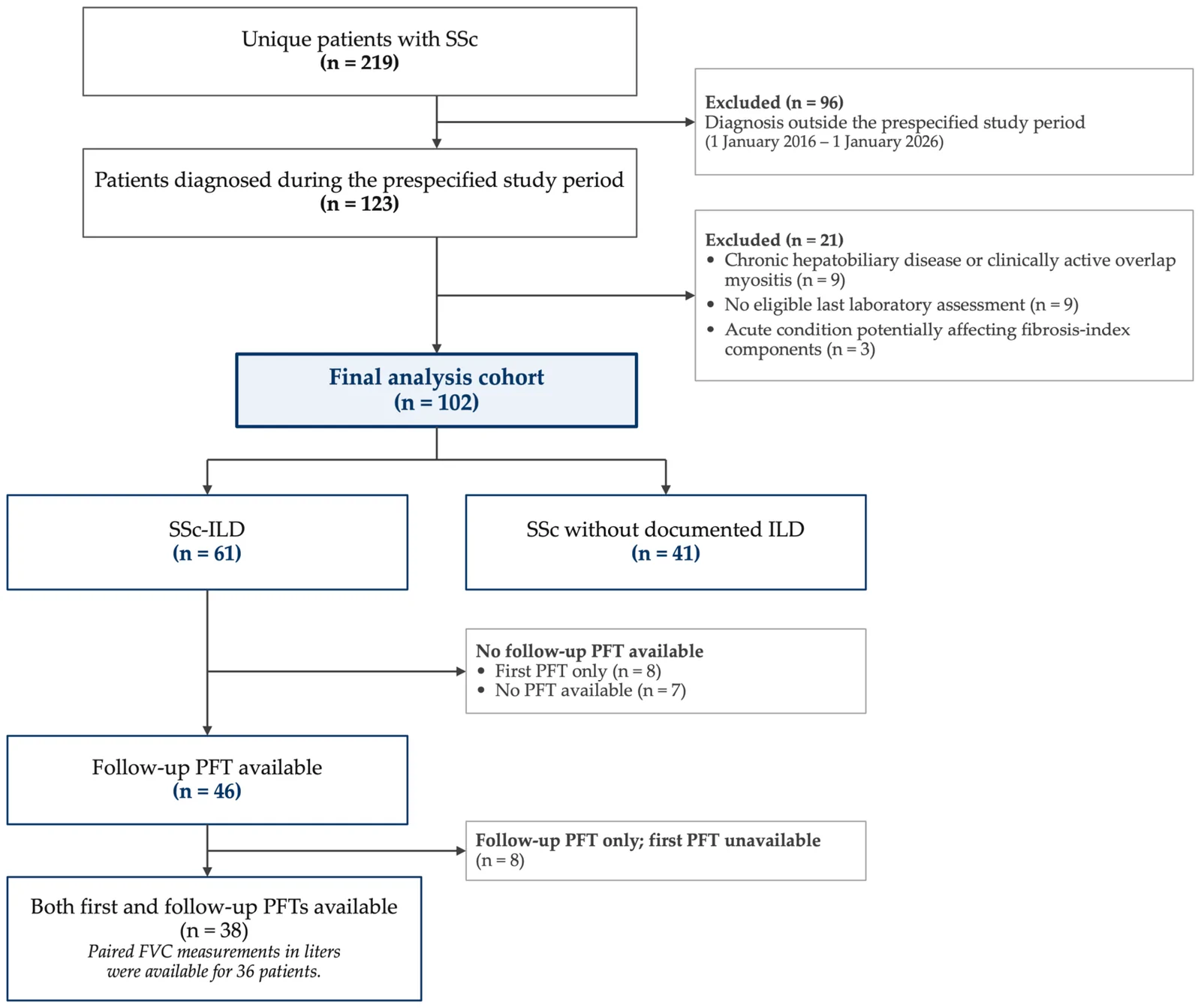
Study flow diagram and definition of the analytical cohorts. Abbreviations: FVC, forced vital capacity; ILD, interstitial lung disease; PFT, pulmonary function test; SSc, systemic sclerosis.

A total of 219 unique patients with SSc were assessed for eligibility. Of these, 96 patients were diagnosed outside the prespecified study period. Among the remaining 123 patients, 21 were excluded because of chronic hepatobiliary disease or clinically active overlap myositis (n = 9), the absence of an eligible laboratory assessment (n = 9), or acute conditions potentially affecting fibrosis-index components (n = 3). The final analytical cohort comprised 102 patients: 61 with documented SSc-ILD and 41 without documented ILD (Figure 1).

The mean age at SSc diagnosis was 47.0 ± 13.8 years, and the mean age at the last clinical assessment was 51.6 ± 13.5 years. Patients with SSc-ILD were older at both diagnosis (49.3 ± 14.0 vs. 43.7 ± 12.8 years, *p* = 0.04) and at the last clinical assessment (53.9 ± 14.0 vs. 48.1 ± 12.0 years, *p* = 0.03) than patients without ILD. The median follow-up duration was similar between groups (51.2 vs. 51.8 months, *p* = 0.87). Women comprised 89.2% of the overall cohort, with no significant difference according to ILD status.

A total of 11 deaths (10.8%) were documented in the available medical records, all among patients with SSc-ILD (11/61, 18.0%). Given the incomplete availability of death dates and non-uniform follow-up, mortality was reported descriptively without inferential comparisons. Diffuse cutaneous SSc was substantially more frequent among patients with ILD, whereas limited cutaneous SSc predominated among those without ILD (*p* < 0.001). Anti-centromere antibody positivity was more common in patients without ILD (51.3% vs. 11.5%, *p* < 0.001), whereas anti-topoisomerase I antibody positivity was more frequent in patients with ILD (67.8% vs. 35.9%, *p* = 0.002). No significant between-group differences were observed in anti-Ro positivity, telangiectasia, Raynaud phenomenon, digital ulcer history, gastrointestinal involvement, or pulmonary hypertension (Table 1).

Among patients with SSc-ILD, 12 of 57 patients (21.1%) with available smoking data had a history of ever smoking. Treatment exposure during follow-up varied according to data availability. Mycophenolate mofetil was the most frequently recorded immunosuppressive therapy [38/50 (76.0%)], followed by cyclophosphamide [16/47 (34.0%)], methotrexate [10/47 (21.3%)], and rituximab [10/47 (21.3%)]. Azathioprine [3/47 (6.4%)] and tocilizumab [3/46 (6.5%)] were used less frequently, and antifibrotic therapy was recorded in 1 of 47 patients (2.1%). Smoking history and available immunosuppressive, antifibrotic, and vasoactive treatment data are summarized in Table 2.

### 3.2. Laboratory Parameters and Non-Invasive Fibrosis Indices

At the last eligible laboratory assessment, patients with SSc-ILD had higher erythrocyte sedimentation rate (ESR) values than those without ILD (median 19.0 vs. 13.0 mm/h, *p* = 0.02), lower hemoglobin levels (12.8 vs. 13.5 g/dL, *p* = 0.02), and higher platelet counts (294.0 vs. 269.0 × 10^9^/L, *p* = 0.01). White blood cell counts were also higher in patients with SSc-ILD (8.3 vs. 6.6 × 10^9^/L, *p* = 0.006), whereas serum creatinine was slightly lower (0.7 vs. 0.8 mg/dL, *p* = 0.04). C-reactive protein (CRP), albumin, ALT, and AST did not differ significantly between groups.

No statistically significant between-group difference was observed for FIB-4 [median 0.9 (IQR 0.6) in both groups, *p* = 0.91] or APRI [median 0.2 (IQR 0.1) in both groups, *p* = 0.06]. Laboratory parameters and fibrosis indices are summarized in Table 3. Exploratory logistic regression analyses of documented SSc-ILD status are presented in Section 3.6 and Supplementary Table S1.

### 3.3. Pulmonary Function Characteristics in SSc-ILD

Among the 61 patients with SSc-ILD, first and follow-up pulmonary function test (PFT) data were available for 46 patients (75.4%) each. Both assessments were available in 38 patients (62.3%), allowing evaluation of longitudinal pulmonary function changes. Eight patients (13.1%) had only a first PFT, eight (13.1%) had only a follow-up PFT, and seven (11.5%) had no available PFT. In an exploratory comparison of patients with and without available follow-up PFT measurements, those with available follow-up PFTs had a longer time since SSc diagnosis at the last assessment (median 63.1 vs. 24.0 months; *p* = 0.029) and higher APRI values (median 0.211 vs. 0.173; *p* = 0.046). Other evaluated characteristics did not differ significantly between these groups (Supplementary Table S2).

At the first available PFT, mean FVC was 87.7 ± 22.3% predicted. At the follow-up PFT, median FVC was 89.0% predicted (IQR 23.5). The last available DLCO was available in 33 patients and had a median value of 67.0% predicted (IQR 26.5).

The median interval from SSc diagnosis to the first available PFT was 3.1 months (IQR 13.3), whereas the median interval from diagnosis to the follow-up PFT was 54.5 months (IQR 62.4). Among patients with both assessments, the median interval between the first and follow-up PFTs was 48.7 months (IQR 60.0). The median absolute interval between the last eligible laboratory assessment and the follow-up PFT was 9.8 months (IQR 10.6). Pulmonary function characteristics and assessment intervals are summarized in Table 4.

### 3.4. Associations of FIB-4 and APRI with Pulmonary Function

In patients with SSc-ILD, neither FIB-4 nor APRI was significantly associated with FVC percentage predicted, DLCO percentage predicted, or FEV1 percentage predicted. FIB-4 showed essentially no association with FVC percentage predicted (ρ = −0.001, *p* = 0.99), and APRI was likewise unassociated with FVC percentage predicted (ρ = 0.027, *p* = 0.86). Similarly, no significant correlations were observed with DLCO percentage predicted (FIB-4: ρ = 0.09, *p* = 0.60; APRI: ρ = 0.078, *p* = 0.67) or FEV1 percentage predicted (FIB-4: ρ = 0.133, *p* = 0.379; APRI: ρ = 0.031, *p* = 0.837).

FIB-4 was inversely correlated with absolute FVC volume (ρ = −0.533, *p* < 0.001), whereas APRI was not significantly associated with absolute FVC volume (ρ = −0.218, *p* = 0.14).

**Figure 2:**
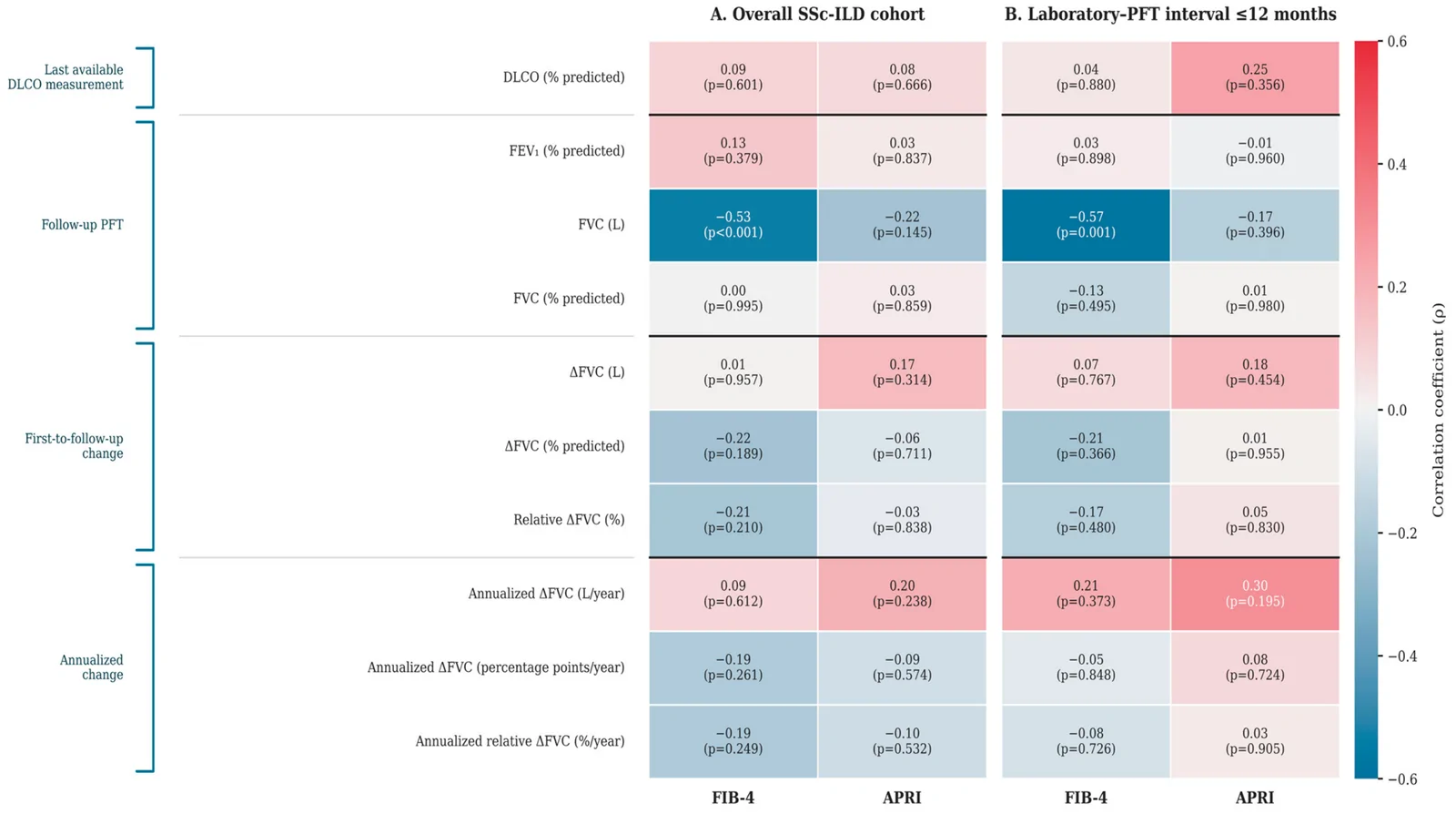
Heatmap of correlations between non-invasive fibrosis indices and pulmonary function parameters in patients with SSc-ILD. Panel (**A**) shows correlations in the overall SSc-ILD cohort, based on available measurements. Panel (**B**) shows the sensitivity analysis restricted to patients with an absolute interval of ≤12 months between the last eligible laboratory assessment and the follow-up PFT. Cell values represent correlation coefficients (ρ), with corresponding *p*-values shown in parentheses. Blue indicates negative correlations and red indicates positive correlations, with color intensity reflecting the magnitude of the correlation coefficient. FEV_1_ and FVC correspond to follow-up PFT measurements, whereas DLCO represents the last available DLCO measurement. Sample sizes vary according to the availability of pulmonary function measurements and are provided in Supplementary Tables S3 and S4. Abbreviations: APRI, aspartate aminotransferase-to-platelet ratio index; DLCO, diffusing capacity of the lung for carbon monoxide; FEV1, forced expiratory volume in 1 s; FIB-4, Fibrosis-4 index; FVC, forced vital capacity; ILD, interstitial lung disease; PFT, pulmonary function test; SSc, systemic sclerosis. Δ indicates first-to-follow-up change.

Neither fibrosis index was significantly associated with first-to-follow-up or annualized changes in FVC. For annualized FVC change expressed as percentage points per year, the correlations were ρ = −0.187 (*p* = 0.26) for FIB-4 and ρ = −0.094 (*p* = 0.57) for APRI. Similarly, no significant associations were observed with relative FVC change or annualized relative FVC change. Supplementary Table S3 presents the full correlation results, and Figure 2 summarizes them visually.

### 3.5. Sensitivity Analysis According to Laboratory–PFT Timing

A sensitivity analysis was performed among patients with an absolute interval of ≤12 months between the last eligible laboratory assessment and the follow-up PFT. Neither FIB-4 nor APRI was associated with FVC percentage predicted (FIB-4: ρ = −0.134, *p* = 0.49; APRI: ρ = 0.005, *p* = 0.98), DLCO percentage predicted (FIB-4: ρ = 0.041, *p* = 0.88; APRI: ρ = 0.247, *p* = 0.36), or annualized FVC change expressed as percentage points per year (FIB-4: ρ = −0.046, *p* = 0.85; APRI: ρ = 0.084, *p* = 0.72).

The inverse association between FIB-4 and absolute FVC volume persisted in this restricted analysis (ρ = −0.572, *p* = 0.001), whereas APRI remained unassociated with absolute FVC volume (ρ = −0.167, *p* = 0.40). Detailed results of the ≤12-month sensitivity analysis are provided in Supplementary Table S4 and Figure 2.

Additional temporal sensitivity analyses examined correlations with follow-up FVC across nested subgroups defined by absolute laboratory–follow-up PFT intervals of ≤3, ≤6, and ≤12 months (n = 11, 14, and 28, respectively; Supplementary Table S5). The inverse correlation between FIB-4 and absolute FVC (L) persisted across all three windows (ρ = −0.845, *p* = 0.001; ρ = −0.829, *p* < 0.001; and ρ = −0.572, *p* = 0.001, respectively).

Within the ≤3-month subgroup, both FIB-4 and APRI showed nominally significant inverse correlations with FVC percentage predicted (ρ = −0.615, *p* = 0.044; and ρ = −0.656, *p* = 0.028, respectively). However, these associations were not statistically significant in the ≤6-month or ≤12-month subgroups. Given the small sample sizes, overlapping patient subsets, and multiple exploratory comparisons, these findings should be interpreted cautiously.

### 3.6. Exploratory and Hypothesis-Generating Logistic and Linear Regression Analyses

Exploratory univariable and multivariable logistic regression analyses were performed to examine the associations of FIB-4 and APRI with documented SSc-ILD status (Supplementary Table S1).

In univariable analyses, neither FIB-4 (OR 0.878, 95% CI 0.420–1.837; *p* = 0.730) nor APRI (OR 0.662 per 0.1-unit increase, 95% CI 0.438–1.001; *p* = 0.051) was significantly associated with ILD status.

In the multivariable models including 96 patients with available cutaneous subtype data, adjustment for age and diffuse cutaneous phenotype revealed a borderline inverse association between FIB-4 and documented ILD status (adjusted odds ratio [aOR] 0.247, 95% CI 0.062–0.988; *p* = 0.048), whereas APRI remained non-significant (aOR 0.635 per 0.1-unit increase, 95% CI 0.393–1.026; *p* = 0.064). Age and diffuse cutaneous phenotype were associated with higher odds of documented ILD in both models.

The borderline FIB-4 association emerged only after adjustment for age, which is itself a component of the FIB-4 formula. This finding warrants cautious interpretation and should not be considered evidence of an independent protective or causal effect.

To further assess potential confounding, exploratory univariable and multivariable linear regression analyses were performed for follow-up FVC percentage predicted and absolute FVC (L) (Supplementary Table S6).

Neither FIB-4 nor APRI was significantly associated with FVC percentage predicted in univariable or age- and sex-adjusted models (FIB-4: adjusted B = −11.44, 95% CI −26.30 to 3.42, *p* = 0.128; APRI: adjusted B = −1.91 per 0.1-unit increase, 95% CI −8.41 to 4.59, *p* = 0.556).

For absolute FVC (L), FIB-4 demonstrated a significant inverse association in the univariable analysis (B = −0.81, 95% CI −1.21 to −0.41, *p* < 0.001). However, this association was attenuated and was no longer statistically significant after adjustment for age and sex (adjusted B = −0.22, 95% CI −0.75 to 0.31, *p* = 0.400). APRI was not significantly associated with absolute FVC in either the univariable or adjusted model (adjusted B = −0.02 per 0.1-unit increase, 95% CI −0.25 to 0.21, *p* = 0.850).

## 4. Discussion

In the present study, we evaluated two widely available non-invasive liver fibrosis indices, FIB-4 and APRI, in relation to ILD status and pulmonary function in patients with SSc. The main finding was no consistent association between either index and pulmonary involvement. FIB-4 and APRI did not differ significantly between patients with documented SSc-ILD and those without documented ILD. Within the SSc-ILD subgroup, neither index showed a statistically significant correlation with FVC percentage predicted, DLCO percentage predicted, or FEV1 percentage predicted. Furthermore, neither FIB-4 nor APRI showed statistically significant correlations with first-to-follow-up or annualized changes in FVC. Collectively, our findings do not support FIB-4 or APRI as reliable standalone indicators of documented ILD status or pulmonary functional impairment in SSc.

Pulmonary function trajectories may not fully capture the complexity of fibrotic lung disease. In SSc-ILD, FVC may remain relatively stable despite declining DLCO, as reported by Le Gouellec et al. [18]. This observation suggests that the absence of FVC decline does not necessarily indicate the absence of pulmonary disease progression [18]. Furthermore, observational data from patients with idiopathic pulmonary fibrosis and progressive pulmonary fibrosis indicate that treatment-related survival differences may not be fully reflected by changes in conventional pulmonary function parameters [19]. However, these findings cannot be directly extrapolated to SSc-ILD, and treatment-related associations remain susceptible to confounding and reverse causation. In our study, the limited availability of serial DLCO measurements, variable intervals between PFT assessments, and lack of standardized imaging follow-up precluded a comprehensive evaluation of pulmonary disease progression.

The exploratory multivariable analyses provided additional insight into the potential influence of confounding. Although neither FIB-4 nor APRI demonstrated a statistically significant unadjusted association with documented SSc-ILD status, FIB-4 showed a borderline inverse association after adjustment for age and diffuse cutaneous phenotype. Importantly, this association was observed in the model adjusted for age and diffuse cutaneous phenotype, but not in the univariable analysis. APRI did not demonstrate a statistically significant association with ILD status after adjustment.

Interpretation of this finding requires particular caution because age is a mathematical component of the FIB-4 formula. Adjusting for age may substantially alter the interpretation of the FIB-4 coefficient, which may reflect variation in aminotransferase levels and platelet counts at a given age rather than differences in underlying fibrotic burden. Older age tends to increase FIB-4, whereas higher platelet counts tend to decrease it. Both characteristics differed between patients with and without documented ILD in our cohort. In contrast, AST and ALT did not differ significantly according to ILD status. Furthermore, the adjusted association was borderline, and the exploratory analyses were not corrected for multiple comparisons. Consequently, this finding should not be interpreted as evidence of a protective effect of FIB-4 or as confirmation of an independent association with documented ILD status.

The rationale for examining liver-derived fibrosis indices in SSc is nevertheless biologically plausible. Fibrotic diseases affecting different organs share core mechanisms, including fibroblast and myofibroblast activation, extracellular matrix accumulation, inflammatory signaling, and tissue remodeling [20]. In addition, hepatic abnormalities and subclinical liver fibrosis have been described in SSc. Lorena et al. evaluated 97 patients with SSc or scleroderma-spectrum disease who underwent transient elastography and identified liver stiffness compatible with clinically relevant fibrosis in a subset of patients [8]. However, increased liver stiffness was associated primarily with conventional hepatic or metabolic factors, including alcohol exposure, waist circumference, alkaline phosphatase levels, and markers of hepatic autoimmunity [8]. Thus, evidence of hepatic involvement in SSc does not necessarily indicate that indirect liver fibrosis indices can quantify fibrotic burden in another organ.

This distinction becomes particularly apparent when comparing FIB-4 and APRI with biomarkers that more directly reflect extracellular matrix turnover. The ELF test combines hyaluronic acid, amino-terminal propeptide of type III procollagen, and tissue inhibitor of metalloproteinase-1. In the original study of 210 patients with SSc, 83% had an ELF score above the normal range. It was associated with skin involvement, disease activity and severity, age, and lower DLCO [4]. These findings were subsequently validated in a European multicenter cohort of 247 patients, in which the ELF score and its components were associated with skin involvement and disease severity, while several components showed inverse associations with FVC, TLC, and DLCO; age, modified Rodnan skin score, and DLCO remained independently associated with ELF score [5]. FIB-4 and APRI differ fundamentally from ELF because they are indirect composite indices derived from routine clinical variables rather than direct measures of extracellular matrix turnover. Accordingly, the absence of associations for FIB-4 and APRI in our cohort should not be interpreted as evidence against circulating fibrosis biomarkers in SSc in general, but rather as evidence that biological specificity may be important when extrapolating a biomarker across organs.

The limited pulmonary specificity of liver-derived fibrosis scores also aligns with the emerging literature on pulmonary fibrosis. Ciornolutchii et al. evaluated FIB-4, APRI, and several other liver fibrosis scores in 38 patients with IPF and 28 patients with secondary pulmonary fibrosis [12]. FIB-4 was higher in IPF than in secondary pulmonary fibrosis. However, the discriminatory ability of hepatic fibrosis scores was limited, and pulmonary function parameters did not differ significantly across hepatic fibrosis risk categories [12]. These findings are broadly consistent with our observation that neither FIB-4 nor APRI was associated with FVC percentage predicted or DLCO percentage predicted. They also suggest that differences in liver-derived scores between pulmonary fibrosis phenotypes, when present, should not automatically be interpreted as evidence that these indices directly quantify the physiological severity of pulmonary fibrosis.

Studies in IPF further suggest that liver fibrosis-related measures may provide prognostic information without necessarily behaving as direct markers of lung function. Cocconcelli et al. identified transient elastography-defined subclinical liver fibrosis in 13 of 37 patients with IPF without known liver disease; liver stiffness was not correlated with clinical-functional parameters, although patients with liver fibrosis had poorer survival, and higher APRI was independently associated with mortality [10]. Similarly, Koyun et al., in a retrospective study of 100 patients with IPF, reported that higher FIB-4 and systemic immune-inflammation index values were independently associated with mortality [11]. However, differences in disease pathobiology, study endpoints, systemic inflammation, comorbidities, and treatment exposures limit the direct extrapolation of these prognostic findings from IPF to SSc-ILD. These associations may reflect a broader systemic risk signal rather than the pulmonary fibrosis burden itself.

A notable finding was the inverse correlation between FIB-4 and absolute FVC volume. FIB-4 correlated with FVC in liters both in the overall SSc-ILD analysis (ρ = −0.533) and in the analysis restricted to a laboratory–PFT interval of ≤12 months (ρ = −0.572), whereas there was essentially no association between FIB-4 and FVC percentage predicted. This discordance argues against interpreting the association with absolute FVC as evidence that FIB-4 directly reflects pulmonary fibrotic severity. FIB-4 incorporates age in its numerator, while absolute FVC is strongly influenced by demographic and anthropometric characteristics, particularly age, sex, and height. Reference equations for spirometry explicitly account for these determinants when deriving expected lung function values [21]. The inverse association between FIB-4 and absolute FVC (L) observed in the unadjusted analyses was attenuated and lost statistical significance after adjustment for age and sex. Nevertheless, residual confounding, particularly by height, cannot be excluded.

The construction of FIB-4 and APRI may also explain their limited association with pulmonary function, as their component variables can vary because of processes unrelated to extracellular matrix deposition in the lung. Furthermore, FIB-4 and APRI perform inconsistently, even within the hepatic diseases for which they were developed. A systematic review supporting the American Association for the Study of Liver Diseases (AASLD) Practice Guideline, including 286 studies and more than 100,000 participants, showed that blood-based fibrosis biomarkers generally resulted in only small-to-moderate changes in the pretest probability of histological fibrosis and that diagnostic performance varied across liver disease etiologies [22]. Extrapolation of these indirect indices from hepatic fibrosis to pulmonary fibrosis therefore introduces an additional layer of biological and methodological uncertainty.

Studies of other circulating markers in SSc-ILD also highlight the importance of biomarker specificity. Elhai et al. evaluated 427 patients with SSc and demonstrated that Krebs von den Lungen-6 (KL-6) correlated with FVC, DLCO, and HRCT-defined lung fibrosis extent. At the same time, C-C motif chemokine ligand 18 (CCL18) was associated with subsequent FVC deterioration and development of extensive disease [23]. More recent studies have similarly linked biomarkers reflecting epithelial injury, immune responses, and matrix remodeling to the presence or progression of SSc-ILD [24]. Conversely, not all mechanistically plausible fibrosis biomarkers are informative: Broens et al. found that circulating fibroblast activation protein levels did not differ between patients with and without SSc-ILD and were not associated with subsequent ILD progression [25]. Most recently, large-scale plasma proteomics identified surfactant protein D (SP-D) as a replicated marker of SSc-ILD and matrix metalloproteinase-8 (MMP-8) as a candidate marker of progressive pulmonary fibrosis across independent cohorts [26]. Together, these studies emphasize that biological plausibility alone is insufficient to establish clinical biomarker validity and that markers more closely linked to pulmonary injury or active fibrogenesis may provide greater disease specificity than generic composite fibrosis indices.

Imaging-based studies further support the distinction between indirect serum scores and direct organ-level assessment. In a recent cross-sectional study, Okyar et al. found higher transient elastography-derived liver stiffness in patients with fibrotic ILD than in healthy controls and associated it with HRCT-defined disease involvement [27]. However, liver stiffness was greater in patients with non-connective tissue disease (non-CTD) fibrotic ILD than in those with connective tissue disease-associated ILD (CTD-ILD). Within the CTD-ILD subgroup, age was the principal variable correlated with liver stiffness [27]. These findings are compatible with cross-organ fibrotic signals but also highlight their phenotype dependence and susceptibility to demographic factors. Importantly, transient elastography measures a physical property of hepatic tissue and should not be regarded as interchangeable with indirect biochemical indices such as FIB-4 and APRI.

The lack of statistically significant correlations between FIB-4 or APRI and historical FVC changes is particularly relevant because serial pulmonary function assessment is an established component of SSc-ILD management. Goh et al. showed that short-term changes in pulmonary function, particularly categorical FVC decline alone or with DLCO decline, were associated with long-term mortality in SSc-ILD [28]. Against this background, the lack of statistically significant associations with first-to-follow-up absolute or relative FVC changes and annualized FVC changes does not establish these indices as reliable markers of functional disease evolution. The main correlation pattern persisted in the ≤12-month sensitivity analysis; however, nominally significant inverse correlations with FVC percentage predicted were observed for both indices in the ≤3-month subgroup (n = 11). Given the small and overlapping subgroups and the multiple exploratory comparisons, these findings require cautious interpretation.

Several strengths of the present study warrant acknowledgment. We evaluated both FIB-4 and APRI in a clinically characterized SSc cohort with and without ILD and excluded major known chronic liver diseases and clinically relevant acute conditions likely to materially affect aminotransferase levels or platelet counts. The indices were analyzed primarily as continuous variables rather than applying hepatic fibrosis thresholds as diagnostic criteria for pulmonary disease. Within SSc-ILD, we examined both cross-sectional pulmonary function and longitudinal FVC change. Furthermore, because laboratory and pulmonary assessments were not uniformly synchronized in this retrospective study, we performed temporal sensitivity analyses across nested absolute laboratory–follow-up PFT intervals of ≤3, ≤6, and ≤12 months to examine the potential influence of assessment timing.

Several limitations should also be considered. First, this retrospective, single-center study included a relatively modest sample without an a priori sample-size calculation, limiting statistical precision and generalizability. Among the 61 patients with SSc-ILD, follow-up PFTs were available for 46 (75.4%), paired FVC percentage-predicted measurements for 38 (62.3%), paired FVC measurements in liters for 36 (59.0%), and DLCO measurements for 33 (54.1%). Patients with available follow-up PFTs had a longer time since SSc diagnosis and higher APRI values than those without, suggesting potential selection differences. However, these exploratory comparisons could not establish the mechanism underlying missingness. Consequently, the available-case analytical subsets may not fully represent the overall SSc-ILD population.

Second, laboratory assessments, HRCT examinations, and PFTs were not performed according to a uniform prospective schedule. The exact dates of initial ILD documentation were unavailable, preventing verification of the temporal relationship between documented ILD status and the laboratory index date. The median absolute laboratory–follow-up PFT interval was 9.8 months, and temporal sensitivity analyses could not eliminate all concerns regarding asynchronous measurements. Furthermore, longitudinal FVC changes were derived from the first and follow-up PFTs and represented annualized two-point changes rather than modeled trajectories. Because FIB-4 and APRI were not measured at a standardized baseline preceding the observation period, these analyses cannot establish their ability to predict incident ILD or subsequent pulmonary function decline.

Third, the multivariable analyses were constrained by the modest sample size and the limited number of covariates that could be included without increasing the risk of overfitting. Residual confounding by clinical characteristics, comorbidities, and treatment exposures cannot be excluded. In particular, age is a component of the FIB-4 formula, complicating interpretation of the age-adjusted association with documented ILD. Regression diagnostics also suggested potential model misfit for APRI and potentially influential observations in the FIB-4 model; additional influence-based sensitivity analyses were not performed. Moreover, height was not included in the adjusted models for absolute FVC volume. Accordingly, the borderline adjusted FIB-4 association should be considered exploratory rather than evidence of a robust independent or protective effect.

Fourth, ILD classification relied on retrospectively available HRCT findings and clinical documentation. Although available HRCT examinations were assessed by an experienced pulmonologist, no independent blinded review or interobserver agreement assessment was performed, and quantitative HRCT fibrosis extent was not analyzed. Importantly, HRCT was not uniformly available among patients classified as having no documented ILD, and the precise extent of missing imaging data could not be quantified. Consequently, undetected subclinical ILD in the comparison group cannot be excluded, and potential verification and disease misclassification bias may have influenced the primary between-group comparisons.

Fifth, FIB-4 and APRI are indirect hepatic fibrosis indices and do not directly measure pulmonary fibrosis. Although recognized chronic liver diseases and relevant acute confounding conditions were excluded, systematic liver elastography or histological assessment was unavailable, and occult hepatic fibrosis could not be excluded. These exclusions may also have narrowed the distributions of the indices. Inflammation-related increases in platelet counts, including potential reactive thrombocytosis, could lower both indices independently of fibrosis burden. Moreover, modified Rodnan skin score (mRSS) and body mass index (BMI) data were unavailable, limiting evaluation of relevant clinical confounders. Treatment-related confounding could not be adequately addressed because comparable treatment information was unavailable for the non-ILD group and cumulative doses, treatment durations, and timing relative to laboratory assessments were inconsistently documented. Confounding by indication also remains possible.

Finally, the numerous exploratory comparisons, including analyses involving multiple pulmonary function parameters and nested temporal sensitivity subgroups, increased the risk of type I error. No adjustment for multiple comparisons was applied, and nominally significant findings, particularly those arising from small subgroups or borderline adjusted associations, should be considered exploratory and hypothesis-generating rather than confirmatory. Conversely, the modest sample size and incomplete measurements may have limited the ability to detect smaller but potentially relevant associations, increasing the possibility of type II error. The absence of external validation further limits the reproducibility and generalizability of these findings. Larger prospective multicenter studies with standardized assessments, prespecified hypotheses, and appropriate sample-size calculations are required.

## 5. Conclusions

In conclusion, our exploratory, hypothesis-generating analyses did not demonstrate consistent associations of FIB-4 and APRI with documented SSc-ILD status, pulmonary function, or historical FVC changes. Although FIB-4 was inversely correlated with absolute FVC volume, this association was no longer statistically significant after adjustment for age and sex, and no corresponding correlation was observed with FVC percentage predicted. These findings suggest that demographic and other non-pulmonary factors may contribute to the observed association. Prospective studies integrating serial FIB-4 and APRI measurements with standardized PFTs, quantitative HRCT, direct assessment of liver fibrosis, and extracellular matrix- or lung-specific biomarkers would help determine whether these readily available indices provide incremental information beyond established pulmonary measures.

## Figures and Tables

**Table 1:** Demographic, clinical, and serological characteristics of patients with systemic sclerosis according to interstitial lung disease status.

	All Patients (n = 102)	SSc Without Documented ILD (n = 41)	SSc with ILD (n = 61)	*p*
Age at SSc diagnosis, years, mean ± SD	47.0 ± 13.8	43.7 ± 12.8	49.3 ± 14.0	0.04
Age at last clinical assessment, years, mean ± SD	51.6 ± 13.5	48.1 ± 12.0	53.9 ± 14.0	0.03
Follow-up duration, months, median (IQR) *	51.7 (58.9)	51.8 (50.2)	51.2 (66.2)	0.87
Sex, female, n (%)	91 (89.2)	35 (85.4)	56 (91.8)	0.34
Documented all-cause mortality, n (%) †	11 (10.8)	0 (0.0)	11 (18.0)	-
SSc subtype				
Diffuse cutaneous SSc, n (%)	41/96 (42.7)	6/36 (16.7)	35/60 (58.3)	<0.001
Limited cutaneous SSc, n (%)	53/96 (55.2)	30/36 (83.3)	23/60 (38.3)	
Sine SSc, n (%)	2/96 (2.1)	0/36 (0.0)	2/60 (3.3)	
ANA positivity, n (%)	94/94 (100.0)	41/41 (100.0)	53/53 (100.0)	—
Anti-centromere Positivity, n (%)	26/91 (28.6)	20/39 (51.3)	6/52 (11.5)	<0.001
Anti-topoisomerase I (Scl-70) positivity, n (%)	54/98 (55.1)	14/39 (35.9)	40/59 (67.8)	0.002
Anti-Ro positivity, n (%)	16/91 (17.6)	7/39 (17.9)	9/52 (17.3)	0.94
Telangiectasia, n (%)	54/102 (52.9)	21/41 (51.2)	33/61 (54.1)	0.77
Raynaud phenomenon, n (%)	97/102 (95.1)	39/41 (95.1)	58/61 (95.1)	1.00
Digital ulcer history, n (%)	33/102 (32.4)	13/41 (31.7)	20/61 (32.8)	0.91
Gastrointestinal involvement, n (%)	46/102 (45.1)	17/41 (41.5)	29/61 (47.5)	0.54
Documented pulmonary hypertension, n (%)	6/94 (6.4)	1/41 (2.4)	5/53 (9.4)	0.23

A dash indicates that no inferential comparison was performed for mortality or that a statistical comparison was not informative because all available ANA results were positive. * Follow-up duration was calculated from the date of SSc diagnosis to the date of death when the exact date of death was available; otherwise, follow-up was calculated to the last recorded clinical visit. † Documented all-cause mortality refers to deaths identified in the available medical records. For patients known to have died but for whom the exact date of death was unavailable, the interval between the last recorded visit and death could not be determined; therefore, mortality should not be interpreted as cumulative mortality over a uniformly observed follow-up period. Percentages were calculated using the available denominator for each variable. Cutaneous subtype data were available for 96 patients; six patients had missing subtype information. Missing data were not imputed. Abbreviations: ANA, antinuclear antibody; ILD, interstitial lung disease; IQR, interquartile range; Scl-70, anti-topoisomerase I antibody; SD, standard deviation; SSc, systemic sclerosis.

**Table 2:** Smoking history and treatment exposures among patients with systemic sclerosis-associated interstitial lung disease.

	SSc with ILD (n = 61)
Smoking, Ever, n (%)	12/57 (21.1)
Treatments received during follow-up, n (%)	
MTX	10/47 (21.3)
AZA	3/47 (6.4)
MMF	38/50 (76.0)
CYC	16/47 (34.0)
TCZ	3/46 (6.5)
RTX	10/47 (21.3)
Antifibrotic therapy	1/47 (2.1)
CCB	6/29 (20.7)
PDE5i	0/29 (0.0)
ERA	1/29 (3.4)

Denominators vary because of missing data and differences in the availability of treatment records. Treatment categories are not mutually exclusive. Treatment data were available for the SSc-ILD subgroup only and could not be systematically compared with the group without documented ILD. Detailed cumulative treatment exposure, treatment duration, and treatment timing relative to the last eligible laboratory assessment were not consistently available; therefore, treatment-related confounding could not be adequately evaluated. Abbreviations: AZA, azathioprine; CCB, calcium channel blocker; CYC, cyclophosphamide; ERA, endothelin receptor antagonist; ILD, interstitial lung disease; MMF, mycophenolate mofetil; MTX, methotrexate; PDE5i, phosphodiesterase-5 inhibitor; RTX, rituximab; SSc, systemic sclerosis; TCZ, tocilizumab.

**Table 3:** Laboratory parameters and non-invasive fibrosis indices according to interstitial lung disease status.

	All Patients (n = 102)	SSc Without Documented ILD (n = 41)	SSc with ILD (n = 61)	*p*
Lab parameters at the last eligible assessment *				
ESR, mm/h, median (IQR)	16.0 (16.5)	13.0 (15.0)	19.0 (16.0)	0.02
CRP, mg/L, median (IQR)	3.2 (5.3)	3.0 (3.3)	3.3 (6.5)	0.08
Hb, g/dL, median (IQR)	13.0 (1.4)	13.5 (1.4)	12.8 (1.5)	0.02
Plt, 10^9^/L, median (IQR)	283.0 (95.2)	269.0 (81.5)	294.0 (121.5)	0.01
WBC, 10^9^/L, median (IQR)	7.6 (2.8)	6.6 (2.0)	8.3 (3.4)	0.006
Alb, g/dL, median (IQR)	4.5 (0.4)	4.5 (0.3)	4.4 (0.4)	0.17
ALT, U/L, median (IQR)	17.0 (10.0)	15.0 (11.5)	18.0 (9.0)	0.45
AST, U/L, median (IQR)	21.0 (8.0)	22.0 (7.5)	20.0 (8.0)	0.70
Cre, mg/dL, median (IQR)	0.7 (0.2)	0.8 (0.2)	0.7 (0.2)	0.04
FIB-4, median (IQR)	0.9 (0.6)	0.9 (0.6)	0.9 (0.6)	0.91
APRI, median (IQR)	0.2 (0.1)	0.2 (0.1)	0.2 (0.1)	0.06

* At the last eligible laboratory assessment, ESR was unavailable in 4/102 patients (3.9%), CRP in 2/102 (2.0%), and albumin in 21/102 (20.6%). The corresponding numbers of missing observations in patients without and with ILD were 2 and 2 for ESR, 1 and 1 for CRP, and 8 and 13 for albumin, respectively. Abbreviations: Alb, albumin; ALT, alanine aminotransferase; APRI, aspartate aminotransferase-to-platelet ratio index; AST, aspartate aminotransferase; Cre, creatinine; CRP, C-reactive protein; ESR, erythrocyte sedimentation rate; FIB-4, Fibrosis-4 index; Hb, hemoglobin; ILD, interstitial lung disease; IQR, interquartile range; Plt, platelet count; SSc, systemic sclerosis; WBC, white blood cell count.

**Table 4:** Pulmonary function characteristics and assessment intervals in patients with systemic sclerosis-associated interstitial lung disease.

	SSc with ILD (n = 61)
First available PFT (n = 46) *	
FEV1, % predicted, mean ± SD	87.7 ± 20.4
FVC, L, median (IQR)	2.5 (1.0)
FVC, % predicted, mean ± SD	87.7 ± 22.3
FEV1/FVC, %, median (IQR)	85.0 (10.5)
Time, from SSc diagnosis to first PFT, months, median (IQR)	3.1 (13.3)
Follow-up PFT (n = 46) *	
FEV1, % predicted, median (IQR)	89.5 (21.5)
FVC, L, Median (IQR)	2.3 (1.2)
FVC, % predicted, median (IQR)	89.0 (23.5)
FEV1/FVC, %, median (IQR)	84.5 (9.2)
Time from SSc diagnosis to follow-up PFT, months, median (IQR)	54.5 (62.4)
Interval between first and follow-up PFTs, months, median (IQR)	48.7 (60.0)
Absolute interval between the last eligible laboratory assessment and the follow-up PFT, months, median (IQR)	9.8 (10.6)
Last available DLCO measurement (n = 33) *	
DLCO, % predicted, median (IQR)	67.0 (26.5)

* Pulmonary function characteristics are presented descriptively for patients with SSc-ILD (n = 61). First and follow-up PFTs were available in 46 patients (75.4%) each, with both assessments available in 38 patients (62.3%). Eight patients had only a first PFT, eight had only a follow-up PFT, and seven had no available PFT. Follow-up PFT measurements were unavailable in 15 patients (24.6%), whereas DLCO measurements were unavailable in 28 patients (45.9%). The interval between the first and follow-up PFTs was calculated only in patients with both assessments (n = 38). Paired FVC measurements were available for 38 patients when expressed as percentage predicted and for 36 patients when expressed in liters. Because the first- and follow-up-PFT groups were not identical, their descriptive values should not be interpreted as paired longitudinal comparisons. DLCO values represented the last available measurements; exact temporal correspondence with the follow-up PFT was not independently verified for all patients. Abbreviations: DLCO, diffusing capacity of the lung for carbon monoxide; FEV1, forced expiratory volume in 1 s; FVC, forced vital capacity; IQR, interquartile range; PFT, pulmonary function test; SD, standard deviation.

## Data Availability

The data presented in this study are available from the corresponding author upon reasonable request, subject to approval by the relevant institutional and ethical authorities. The data are not publicly available due to patient privacy and ethical restrictions.

## Supplementary Materials

The following supporting information can be downloaded at https://www.mdpi.com/article/10.3390/diagnostics16213575/s1: Table S1: Univariable and multivariable logistic regression analyses of documented SSc-ILD status; Table S2: Comparison of patients with SSc-ILD according to follow-up pulmonary function test availability; Table S3: Correlations between FIB-4, APRI, and pulmonary function parameters in patients with SSc-ILD; Table S4: Correlations between fibrosis indices and pulmonary function parameters in patients with SSc-ILD with a laboratory–follow-up PFT interval of ≤12 months; Table S5: Temporal sensitivity analyses of correlations between fibrosis indices and follow-up FVC across laboratory–PFT intervals of ≤3, ≤6, and ≤12 months; Table S6: Univariable and multivariable linear regression analyses of follow-up FVC in patients with SSc-ILD; Supplementary Code S1: Python (version 3.13.18; Google Colab environment) code used to generate the correlation heatmaps shown in Figure 2.

## Abbreviations

AASLD	American Association for the Study of Liver Diseases
ACR/EULAR	American College of Rheumatology/European League Against Rheumatism
Alb	Albumin
ALT	Alanine aminotransferase
ANA	Antinuclear antibody
APRI	Aspartate aminotransferase-to-platelet ratio index
AST	Aspartate aminotransferase
AZA	Azathioprine
CCB	Calcium channel blocker
CCL18	C-C motif chemokine ligand 18
Cre	Creatinine
CRP	C-reactive protein
CTD-ILD	Connective tissue disease-associated interstitial lung disease
CYC	Cyclophosphamide
dcSSc	Diffuse cutaneous systemic sclerosis
DLCO	Diffusing capacity of the lung for carbon monoxide
ELF	Enhanced Liver Fibrosis
ERA	Endothelin receptor antagonist
ESR	Erythrocyte sedimentation rate
FEV1	Forced expiratory volume in 1 s
FIB-4	Fibrosis-4 index
FVC	Forced vital capacity
Hb	Hemoglobin
HRCT	High-resolution computed tomography
ILD	Interstitial lung disease
IPF	Idiopathic pulmonary fibrosis
IQR	Interquartile range
KL-6	Krebs von den Lungen-6
lcSSc	Limited cutaneous systemic sclerosis
MMP-8	Matrix metalloproteinase-8
MMF	Mycophenolate mofetil
MTX	Methotrexate
PDE5i	Phosphodiesterase-5 inhibitor
PFT	Pulmonary function test
Plt	Platelet count
RTX	Rituximab
Scl-70	Anti-topoisomerase I antibody
SD	Standard deviation
SP-D	Surfactant protein D
SSc	Systemic sclerosis
SSc-ILD	Systemic sclerosis-associated interstitial lung disease
TCZ	Tocilizumab
TLC	Total lung capacity
ULN	Upper limit of normal
WBC	White blood cell count

## References

[1] Denton C.P., Khanna D. (2017). Systemic sclerosis. Lancet.

[2] Hoffmann-Vold A.M., Maher T.M., Philpot E.E., Ashrafzadeh A., Barake R., Barsotti S., Bruni C., Carducci P., Carreira P.E., Castellvi I. (2020). The identification and management of interstitial lung disease in systemic sclerosis: Evidence-based European consensus statements. Lancet Rheumatol..

[3] Raghu G., Montesi S.B., Silver R.M., Hossain T., Macrea M., Herman D., Barnes H., Adegunsoye A., Azuma A., Chung L. (2024). Treatment of Systemic Sclerosis-associated Interstitial Lung Disease: Evidence-based Recommendations. An Official American Thoracic Society Clinical Practice Guideline. Am. J. Respir. Crit. Care Med..

[4] Abignano G., Cuomo G., Buch M.H., Rosenberg W.M., Valentini G., Emery P., Del Galdo F. (2014). The enhanced liver fibrosis test: A clinical grade, validated serum test, biomarker of overall fibrosis in systemic sclerosis. Ann. Rheum. Dis..

[5] Abignano G., Blagojevic J., Bissell L.A., Dumitru R.B., Eng S., Allanore Y., Avouac J., Bosello S., Denton C.P., Distler O. (2019). European multicentre study validates enhanced liver fibrosis test as biomarker of fibrosis in systemic sclerosis. Rheumatology.

[6] Sterling R.K., Lissen E., Clumeck N., Sola R., Correa M.C., Montaner J., Sulkowski M.S., Torriani F.J., Dieterich D.T., Thomas D.L. (2006). Development of a simple noninvasive index to predict significant fibrosis in patients with HIV/HCV coinfection. Hepatology.

[7] Wai C.T., Greenson J.K., Fontana R.J., Kalbfleisch J.D., Marrero J.A., Conjeevaram H.S., Lok A.S. (2003). A simple noninvasive index can predict both significant fibrosis and cirrhosis in patients with chronic hepatitis C. Hepatology.

[8] Lorena M., Bellan M., Lepore M., Sola D., Pedrazzoli R., Rigamonti C., De Benedittis C., Manfredi G.F., Acquaviva A., Tonello S. (2022). Clinical Relevance of Liver Involvement in the Clinical Course of Systemic Sclerosis. J. Clin. Med..

[9] Lee S.W., Kim B.K., Park J.Y., Kim D.Y., Ahn S.H., Song J.J., Park Y.B., Lee S.K., Han K.H., Kim S.U. (2015). Disease duration and Medsger’s severity score are associated with significant liver fibrosis in patients with systemic sclerosis. Clin. Exp. Rheumatol..

[10] Cocconcelli E., Tonelli R., Abbati G., Marchioni A., Castaniere I., Pelizzaro F., Russo F.P., Vegetti A., Balestro E., Pietrangelo A. (2021). Subclinical liver fibrosis in patients with idiopathic pulmonary fibrosis. Intern. Emerg. Med..

[11] Koyun G.B., Berk S., Dogan O.T. (2024). The importance of SII and FIB-4 scores in predicting mortality in idiopathic pulmonary fibrosis patients. Clin. Biochem..

[12] Ciornolutchii V., Ruta V.M., Man A.M., Motoc N.S., Popa S.L., Dumitrascu D.L., Ismaiel A., Leucuta D.C. (2024). Exploring the Role of Hemogram-Derived Ratios and Liver Fibrosis Scores in Pulmonary Fibrosis. Medicina.

[13] van den Hoogen F., Khanna D., Fransen J., Johnson S.R., Baron M., Tyndall A., Matucci-Cerinic M., Naden R.P., Medsger T.A., Carreira P.E. (2013). 2013 classification criteria for systemic sclerosis: An American college of rheumatology/European league against rheumatism collaborative initiative. Ann. Rheum. Dis..

[14] Vasi İ., Karaduman İ., Karaca Ö.O., Duran R., Kardaş R.C., Çakir İ.Y., Eseroğlu E., Karakaya B., Bölek E.Ç., Küçük H. (2026). Interstitial lung disease in systemic sclerosis: Beyond respiratory failure to the hidden iceberg of mortality. Rheumatol. Q..

[15] Vasi I., Karaduman I., Alacaoglu S., Duygulu S., Kardas R.C., Yildirim D., Kaya B., Duran R., Cakir I.Y., Eseroglu E. (2026). Assessment of interstitial lung disease in systemic sclerosis using oscillometric methods: A novel approach to respiratory assessment. Rheumatology.

[16] LeRoy E.C., Black C., Fleischmajer R., Jablonska S., Krieg T., Medsger T.A., Rowell N., Wollheim F. (1988). Scleroderma (systemic sclerosis): Classification, subsets and pathogenesis. J. Rheumatol..

[17] Graham B.L., Steenbruggen I., Miller M.R., Barjaktarevic I.Z., Cooper B.G., Hall G.L., Hallstrand T.S., Kaminsky D.A., McCarthy K., McCormack M.C. (2019). Standardization of Spirometry 2019 Update. An Official American Thoracic Society and European Respiratory Society Technical Statement. Am. J. Respir. Crit. Care Med..

[18] Le Gouellec N., Duhamel A., Perez T., Hachulla A.L., Sobanski V., Faivre J.B., Morell-Dubois S., Lambert M., Hatron P.Y., Hachulla E. (2017). Predictors of lung function test severity and outcome in systemic sclerosis-associated interstitial lung disease. PLoS ONE.

[19] Andrisano A.G., Castaldo N., Giuliana F., Femia D., Morana G., Patruno V., Monteleone G., Reccardini N., Cifaldi R., Hughes M. (2025). Retrospective Observational Study of Nintedanib in Managing Idiopathic and Progressive Pulmonary Fibrosis in Routine Practice. J. Clin. Med..

[20] Rieder F., Nagy L.E., Maher T.M., Distler J.H.W., Kramann R., Hinz B., Prunotto M. (2025). Fibrosis: Cross-organ biology and pathways to development of innovative drugs. Nat. Rev. Drug Discov..

[21] Quanjer P.H., Stanojevic S., Cole T.J., Baur X., Hall G.L., Culver B.H., Enright P.L., Hankinson J.L., Ip M.S., Zheng J. (2012). Multi-ethnic reference values for spirometry for the 3-95-yr age range: The global lung function 2012 equations. Eur. Respir. J..

[22] Patel K., Asrani S.K., Fiel M.I., Levine D., Leung D.H., Duarte-Rojo A., Dranoff J.A., Nayfeh T., Hasan B., Taddei T.H. (2025). Accuracy of blood-based biomarkers for staging liver fibrosis in chronic liver disease: A systematic review supporting the AASLD Practice Guideline. Hepatology.

[23] Elhai M., Hoffmann-Vold A.M., Avouac J., Pezet S., Cauvet A., Leblond A., Fretheim H., Garen T., Kuwana M., Molberg O. (2019). Performance of Candidate Serum Biomarkers for Systemic Sclerosis-Associated Interstitial Lung Disease. Arthritis Rheumatol..

[24] Gyorfi A.H., Filla T., Dickel N., Moller F., Li Y.N., Bergmann C., Matei A.E., Harrer T., Kunz M., Schett G. (2024). Performance of serum biomarkers reflective of different pathogenic processes in systemic sclerosis-associated interstitial lung disease. Rheumatology.

[25] Broens B., van der Laken C.J., Simons I.A., Dekker T., Duitman J.W., Voskuyl A.E. (2025). Longitudinal assessment of circulating fibroblast activation protein in systemic sclerosis-associated interstitial lung disease. Arthritis Res. Ther..

[26] Gerges E., Cauvet A., Guedot M., Dahan M., Mairet M., Avouac J., Gobeaux C., Gautier E.F., Gall M.L., Clary G. (2026). High-throughput plasma proteomics reveals circulating biomarkers of interstitial lung disease and progressive pulmonary fibrosis in systemic sclerosis. Ann. Rheum. Dis..

[27] Okyar B., Yuce S., Karacan E., Arici F.N., Yildirim A., Koc E., Tuzun Z., Kara S., Dilek O., Sumbul H.E. (2025). Increased transient elastography-derived liver stiffness in fibrotic interstitial lung disease compared with healthy controls and its association with high-resolution computed tomography extent: A cross-sectional observational study. Rheumatol. Int..

[28] Goh N.S., Hoyles R.K., Denton C.P., Hansell D.M., Renzoni E.A., Maher T.M., Nicholson A.G., Wells A.U. (2017). Short-Term Pulmonary Function Trends Are Predictive of Mortality in Interstitial Lung Disease Associated With Systemic Sclerosis. Arthritis Rheumatol..

