# Supplementary material for: Do Non-Invasive Fibrosis Indices Reflect Interstitial Lung Disease and Pulmonary Function in Systemic Sclerosis?
Source: Diagnostics (Basel). 2026 Oct 29;16(21):3575. doi: 10.3390/diagnostics16213575 (PMC13762115; doi:10.3390/diagnostics16213575)
Supplement: Supplementary file 1 [file diagnostics-16-03575-s001.zip › diagnostics-4611696-supplementary.pdf]

Supplementary Materials

Table S1. Univariable and multivariable logistic regression analyses of factors associated with documented SSc-ILD status.

Table S2. Comparison of demographic and clinical characteristics and non-invasive fibrosis indices according to follow-up PFT availability among patients with SSc-ILD.

Table S3. Correlations between non-invasive fibrosis indices and pulmonary function parameters in patients with SSc-ILD.

Table S4. Sensitivity analysis of correlations between non-invasive fibrosis indices and pulmonary function parameters in patients with SSc-ILD whose follow-up PFT was performed within 12 months of the last eligible laboratory assessment.

Table S5. Additional sensitivity analyses of correlations between non-invasive fibrosis indices and follow-up FVC for laboratory–PFT intervals of ≤3, ≤6, and ≤12 months in patients with SSc-ILD.

Table S6. Univariable and multivariable linear regression analyses of associations between non-invasive fibrosis indices and follow-up FVC (in liters and percentage predicted) in patients with SSc-ILD.

Code S1. Python code used to generate the correlation heatmaps shown in Figure 2.

Table S1. Univariable and multivariable logistic regression analyses of factors associated with documented SSc-ILD status.

Variable	Univariable OR (95% CI)	p	FIB-4 model aOR (95% CI)	p	APRI model aOR (95% CI)	p
FIB-4, per 1-unit increase	0.878 (0.420–1.837)	0.730	0.247 (0.062–0.988)	0.048	—	—
APRI, per 0.1-unit increase	0.662 (0.438–1.001)	0.051	—	—	0.635 (0.393–1.026)	0.064
Age, per 10-year increase	1.394 (1.024–1.898)	0.035	2.505 (1.407–4.461)	0.002	1.814 (1.204–2.734)	0.004
Diffuse cutaneous phenotype	7.000 (2.535–19.330)	<0.001	8.754 (2.838–27.006)	<0.001	7.939 (2.603–24.210)	<0.001

Univariable analyses included 102 patients for FIB-4, APRI, and age, and 96 patients for diffuse cutaneous phenotype. Both multivariable models included 96 patients (60 with ILD and 36 without ILD). FIB-4 and APRI were evaluated in separate models adjusted for age at the last eligible laboratory assessment and diffuse cutaneous phenotype. The reference category for diffuse cutaneous phenotype was non-diffuse SSc, comprising limited cutaneous SSc and sine scleroderma. All analyses were exploratory, and no adjustment for multiple comparisons was applied.
Abbreviations: APRI, aspartate aminotransferase-to-platelet ratio index; aOR, adjusted odds ratio; CI, confidence interval; FIB-4, Fibrosis-4 index; OR, odds ratio; SSc-ILD, systemic sclerosis-associated interstitial lung disease.

Table S2. Comparison of demographic and clinical characteristics and non-invasive fibrosis indices according to follow-up PFT availability among patients with SSc-ILD.

Characteristic	Follow-up PFT unavailable (n = 15)	Follow-up PFT available (n = 46)	p
Age at last assessment, years	56.71 (13.34)	55.70 (23.07)	0.893
Time since SSc diagnosis at last assessment, months	23.98 (53.45)	63.13 (70.02)	0.029
FIB-4	0.785 (0.37)	0.902 (0.67)	0.375
APRI	0.173 (0.09)	0.211 (0.09)	0.046
Female sex, n (%)	14 (93.3)	42 (91.3)	1.000
Diffuse cutaneous SSc, n/N (%)	9/14 (64.3)	26/46 (56.5)	0.760

Continuous variables are presented as median (interquartile range), and categorical variables as n (%) or n/N (%), as appropriate. Continuous variables were compared using the Mann–Whitney U test, and categorical variables using Fisher's exact test. Among patients without follow-up PFT data (n = 15), eight had only an first available PFT and seven had no available PFT. Among those with follow-up PFT data (n = 46), 38 had both first available and follow-up assessments and eight had only a follow-up assessment. Cutaneous subtype was available for 60 of 61 patients; one patient in the group without follow-up PFT data had missing subtype information. Diffuse cutaneous SSc was compared with non-diffuse SSc (limited cutaneous SSc and sine scleroderma). All comparisons were exploratory, and no adjustment for multiple comparisons was applied.
Abbreviations: APRI, aspartate aminotransferase-to-platelet ratio index; FIB-4, Fibrosis-4 index; PFT, pulmonary function test; SSc, systemic sclerosis; SSc-ILD, systemic sclerosis-associated interstitial lung disease.

Table S3. Correlations between non-invasive fibrosis indices and pulmonary function parameters in patients with SSc-ILD.

Pulmonary function parameters	FIB-4			APRI		
	ρ	*p*	n	ρ	*p*	n
**Last available DLCO measurement**						
DLCO, % predicted	0.094	0.601	33	0.078	0.666	33
**Follow-up PFT**						
FEV1, % predicted	0.133	0.379	46	0.031	0.837	46
FVC, L	−0.533	<0.001	46	−0.218	0.145	46
FVC, % predicted	−0.001	0.995	46	0.027	0.859	46
**First-to-follow-up change**						
ΔFVC, L	0.009	0.957	36	0.173	0.314	36
ΔFVC, % predicted	−0.218	0.189	38	−0.062	0.711	38
Relative FVC change, %	−0.208	0.210	38	−0.034	0.838	38
**Annualized change**						
Annualized ΔFVC, L/year	0.088	0.612	36	0.202	0.238	36
Annualized ΔFVC, percentage points/year	−0.187	0.261	38	−0.094	0.574	38
Annualized relative ΔFVC, %/year	−0.192	0.249	38	−0.105	0.532	38

Data are presented as Spearman's rank correlation coefficients (ρ), with corresponding p-values and sample sizes (n). Sample sizes varied according to the availability of pulmonary function measurements. Follow-up FEV_1 and FVC measurements were available for 46 patients, whereas the last available DLCO measurement was available for 33 patients. Paired FVC measurements were available for 38 patients when expressed as percentage predicted and for 36 patients when expressed in liters. Longitudinal FVC changes were calculated from the first available and follow-up PFT measurements. All analyses were exploratory, and no adjustment for multiple comparisons was applied.
Abbreviations: APRI, aspartate aminotransferase-to-platelet ratio index; DLCO, diffusing capacity of the lung for carbon monoxide; FEV_1, forced expiratory volume in one second; FIB-4, Fibrosis-4 index; FVC, forced vital capacity; PFT, pulmonary function test; SSc-ILD, systemic sclerosis-associated interstitial lung disease.

Table S4. Sensitivity analysis of correlations between non-invasive fibrosis indices and pulmonary function parameters in patients with SSc-ILD whose follow-up PFT was performed within 12 months of the last eligible laboratory assessment.

**Pulmonary function test parameters**	**FIB-4**			**APRI**		
	**ρ**	***p***	**n**	**ρ**	***p***	**n**
**Last available DLCO measurement**						
DLCO, % predicted	0.041	0.880	16	0.247	0.356	16
**Follow-up PFT**						
FEV1, % predicted	0.025	0.898	28	−0.010	0.960	28
FVC, L	−0.572	0.001	28	−0.167	0.396	28
FVC, % predicted	−0.134	0.495	28	0.005	0.980	28
**First-to-follow-up change**						
ΔFVC, L	0.071	0.767	20	0.177	0.454	20
ΔFVC, % predicted	−0.214	0.366	20	0.014	0.955	20
Relative FVC change, %	−0.168	0.480	20	0.051	0.830	20
**Annualized change**						
Annualized ΔFVC, L/year	0.211	0.373	20	0.302	0.195	20
Annualized ΔFVC, percentage points/year	−0.046	0.848	20	0.084	0.724	20
Annualized relative ΔFVC, %/year	−0.083	0.726	20	0.029	0.905	20

Data are presented as Spearman's rank correlation coefficients (ρ), with corresponding p-values and sample sizes (n). The sensitivity analysis was restricted to patients with an absolute interval of 12 months or less between the last eligible laboratory assessment and the follow-up PFT. The same restriction was applied to analyses of historical first-to-follow-up and annualized FVC changes. DLCO was generally measured during the same pulmonary function assessment, although exact temporal alignment was not independently verified for all patients. Sample sizes varied according to the availability of pulmonary function measurements. All analyses were exploratory, and no adjustment for multiple comparisons was applied.

Abbreviations: APRI, aspartate aminotransferase-to-platelet ratio index; DLCO, diffusing capacity of the lung for carbon monoxide; FEV_1, forced expiratory volume in one second; FIB-4, Fibrosis-4 index; FVC, forced vital capacity; PFT, pulmonary function test; SSc-ILD, systemic sclerosis-associated interstitial lung disease.

Table S5. Additional sensitivity analyses of correlations between non-invasive fibrosis indices and follow-up FVC for laboratory–PFT intervals of ≤3, ≤6, and ≤12 months in patients with SSc-ILD.

Laboratory–PFT interval	Index	n	FVC (L), ρ (p)	FVC (% predicted), ρ (p)
≤3 months	FIB-4	11	−0.845 (0.001)	−0.615 (0.044)
	APRI	11	−0.218 (0.519)	−0.656 (0.028)
≤6 months	FIB-4	14	−0.829 (<0.001)	−0.370 (0.193)
	APRI	14	−0.319 (0.266)	−0.407 (0.149)
≤12 months	FIB-4	28	−0.572 (0.001)	−0.134 (0.495)
	APRI	28	−0.167 (0.396)	0.005 (0.980)

Time windows were defined according to the absolute interval between the last eligible laboratory assessment and the follow-up PFT. The intervals represent nested patient subsets rather than mutually exclusive groups. Associations were evaluated using Spearman's rank correlation analysis. The ≤12-month results are also presented in Table S4 as part of the broader pulmonary function sensitivity analysis. All analyses were exploratory and unadjusted, and no correction for multiple comparisons was applied. Findings from the smaller subsets should be interpreted cautiously.

Abbreviations: APRI, aspartate aminotransferase-to-platelet ratio index; FIB-4, Fibrosis-4 index; FVC, forced vital capacity; PFT, pulmonary function test; SSc-ILD, systemic sclerosis-associated interstitial lung disease; ρ, Spearman's rank correlation coefficient.

Table S6. Univariable and multivariable linear regression analyses of associations between non-invasive fibrosis indices and follow-up FVC (in liters and percentage predicted) in patients with SSc-ILD.

Predictor	Univariable B (95% CI)	p	Adjusted B (95% CI)	p
**Outcome: FVC (% predicted)**				
FIB-4, per 1-unit increase	−4.77 (−15.26 to 5.73)	0.365	−11.44 (−26.30 to 3.42)	0.128
APRI, per 0.1-unit increase	−0.65 (−6.81 to 5.52)	0.833	−1.91 (−8.41 to 4.59)	0.556
**Outcome: FVC (L)**				
FIB-4, per 1-unit increase	−0.81 (−1.21 to −0.41)	<0.001	−0.22 (−0.75 to 0.31)	0.400
APRI, per 0.1-unit increase	−0.21 (−0.48 to 0.06)	0.122	−0.02 (−0.25 to 0.21)	0.850

All analyses included 46 patients with available follow-up FVC measurements. FIB-4 and APRI were evaluated in separate models. Multivariable models were adjusted for age at the last eligible laboratory assessment and sex. Regression coefficients represent differences in FVC percentage points or liters, according to the outcome, per specified unit increase in the fibrosis index. All analyses were exploratory, and no adjustment for multiple comparisons was applied.

Abbreviations: B, unstandardized regression coefficient; CI, confidence interval; FIB-4, Fibrosis-4 index; APRI, aspartate aminotransferase-to-platelet ratio index; FVC, forced vital capacity; SSc-ILD, systemic sclerosis-associated interstitial lung disease.

Code S1. Python code used to generate the correlation heatmaps shown in Figure 2.

The code was executed in Google Colab using the pandas, seaborn, and matplotlib libraries. Correlation coefficients and corresponding p-values were calculated separately using IBM SPSS Statistics version 24.0. Python was used exclusively to visualize these previously calculated results and was not used to perform the statistical analyses.

```
# ============================================================
# Code S1
# Python code used to generate the correlation heatmaps
# shown in Figure 2.
#
# Correlation coefficients and p-values were calculated
# separately using IBM SPSS Statistics version 24.0.
# Python is used exclusively for visualization.
#
# Compatible with Google Colab.
# ============================================================

import pandas as pd
import seaborn as sns
import matplotlib.pyplot as plt
import matplotlib.font_manager as fm


# ============================================================
# 1. Font setup
# ============================================================

available_fonts = set(
    font.name for font in fm.fontManager.ttflist
)

if "Palatino Linotype" in available_fonts:
    selected_font = "Palatino Linotype"
elif "TeX Gyre Pagella" in available_fonts:
```

```
    selected_font = "TeX Gyre Pagella"
else:
    selected_font = "DejaVu Serif"

sns.set_theme(
    style="white",
    rc={
        "font.family": [selected_font, "DejaVu Serif"],
        "font.size": 10
    }
)

print("Selected font:", selected_font)


# ============================================================
# 2. Pulmonary function parameter order
# ============================================================

# Four analytical groups:
#
# Group 1: Last available DLCO measurement (row 0)
# Group 2: Follow-up PFT (rows 1-3)
# Group 3: First-to-follow-up change (rows 4-6)
# Group 4: Annualized change (rows 7-9)

parameters = [
    "DLCO (% predicted)",
    "FEV₁ (% predicted)",
    "FVC (L)",
    "FVC (% predicted)",
    "ΔFVC (L)",
    "ΔFVC (% predicted)",
    "Relative ΔFVC (%)",
    "Annualized ΔFVC (L/year)",
    "Annualized ΔFVC (percentage points/year)",
```

```
    "Annualized relative ΔFVC (%/year)"
]


# ==================================================================
# 3. Overall SSc-ILD cohort
# ==================================================================

overall = pd.DataFrame({
    "Parameter": parameters,

    "FIB-4_rho": [
        0.094,
        0.133,
        -0.533,
        -0.001,
        0.009,
        -0.218,
        -0.208,
        0.088,
        -0.187,
        -0.192
    ],

    "FIB-4_p": [
        "0.601",
        "0.379",
        "<0.001",
        "0.995",
        "0.957",
        "0.189",
        "0.210",
        "0.612",
        "0.261",
        "0.249"
    ],
```

```
    "APRI_rho": [
        0.078,
        0.031,
        -0.218,
        0.027,
        0.173,
        -0.062,
        -0.034,
        0.202,
        -0.094,
        -0.105
    ],

    "APRI_p": [
        "0.666",
        "0.837",
        "0.145",
        "0.859",
        "0.314",
        "0.711",
        "0.838",
        "0.238",
        "0.574",
        "0.532"
    ]
})


# ============================================================
# 4. Sensitivity analysis:
#    laboratory–follow-up PFT interval <=12 months
# ============================================================

sensitivity = pd.DataFrame({
    "Parameter": parameters,
```

"FIB-4_rho": [
0.041,
0.025,
-0.572,
-0.134,
0.071,
-0.214,
-0.168,
0.211,
-0.046,
-0.083
],

"FIB-4_p": [
"0.880",
"0.898",
"0.001",
"0.495",
"0.767",
"0.366",
"0.480",
"0.373",
"0.848",
"0.726"
],

"APRI_rho": [
0.247,
-0.010,
-0.167,
0.005,
0.177,
0.014,
0.051,
0.302,

```
        0.084,
        0.029
    ],

    "APRI_p": [
        "0.356",
        "0.960",
        "0.396",
        "0.980",
        "0.454",
        "0.955",
        "0.830",
        "0.195",
        "0.724",
        "0.905"
    ]
})


# ================================================================
# 5. Formatting functions
# ================================================================

def format_rho(rho):
    """
    Display correlation coefficients to two decimal places.
    Avoid negative zero such as -0.00.
    """
    display_value = 0.0 if abs(rho) < 0.005 else rho
    return f"{display_value:.2f}"


def format_p(p):
    """
    Preserve p-values exactly as supplied from SPSS.
    """
```

```
    p = str(p)

    if p.startswith("<"):
        return f"p{p}"

    return f"p={p}"


def prepare_heatmap(df):
    """
    Construct the correlation matrix and annotation labels.
    """

    rho_matrix = pd.DataFrame(
        {
            "FIB-4": df["FIB-4_rho"].values,
            "APRI": df["APRI_rho"].values
        },
        index=df["Parameter"]
    )

    labels = pd.DataFrame(
        index=df["Parameter"]
    )

    labels["FIB-4"] = [
        f"{format_rho(rho)}\n({format_p(p)})"
        for rho, p in zip(
            df["FIB-4_rho"],
            df["FIB-4_p"]
        )
    ]

    labels["APRI"] = [
        f"{format_rho(rho)}\n({format_p(p)})"
        for rho, p in zip(
```

```
            df["APRI_rho"],
            df["APRI_p"]
        )
    ]

    return rho_matrix, labels


overall_matrix, overall_labels = prepare_heatmap(overall)

sensitivity_matrix, sensitivity_labels = prepare_heatmap(
    sensitivity
)


# ============================================================
# 6. Figure layout
#
# Separate axes are reserved for:
#   1. Analytical group headings and brackets
#   2. Pulmonary function parameter labels
#   3. Overall-cohort heatmap
#   4. Sensitivity-analysis heatmap
#   5. Color bar
#
# This prevents group headings from overlapping
# pulmonary function parameter labels.
# ============================================================

fig = plt.figure(
    figsize=(16.5, 8.8),
    facecolor="white"
)

grid = fig.add_gridspec(
    nrows=1,
```

```
    ncols=5,

    width_ratios=[
        1.6,     # Analytical groups
        3.9,     # Parameter names
        2.9,     # Panel A
        2.9,     # Panel B
        0.20     # Color bar
    ],

    left=0.03,
    right=0.975,
    top=0.90,
    bottom=0.12,
    wspace=0.055
)

ax_groups = fig.add_subplot(grid[0, 0])
ax_labels = fig.add_subplot(grid[0, 1])
ax_overall = fig.add_subplot(grid[0, 2])
ax_sensitivity = fig.add_subplot(grid[0, 3])
ax_colorbar = fig.add_subplot(grid[0, 4])


# ==================================================================
# 7. Heatmap color palette
# ==================================================================

# Blue = negative correlation
# White = correlation near zero
# Red = positive correlation

cmap = sns.diverging_palette(
    240,
    10,
    s=80,
```

```
    l=45,
    as_cmap=True
)


# ================================================================
# 8. Panel A: Overall SSc-ILD cohort
# ================================================================

sns.heatmap(
    overall_matrix,

    annot=overall_labels,
    fmt="",

    cmap=cmap,
    center=0,
    vmin=-0.60,
    vmax=0.60,

    linewidths=1.2,
    linecolor="white",

    xticklabels=True,
    yticklabels=False,

    cbar=False,

    annot_kws={
        "fontsize": 9.3
    },

    ax=ax_overall
)
```

```
# ==========================================================
# 9. Panel B: Sensitivity analysis
# ==========================================================

sns.heatmap(
    sensitivity_matrix,

    annot=sensitivity_labels,
    fmt="",

    cmap=cmap,
    center=0,
    vmin=-0.60,
    vmax=0.60,

    linewidths=1.2,
    linecolor="white",

    xticklabels=True,
    yticklabels=False,

    cbar=True,
    cbar_ax=ax_colorbar,

    cbar_kws={
        "ticks": [
            -0.6,
            -0.4,
            -0.2,
            0,
            0.2,
            0.4,
            0.6
        ]
    },
```

```
    annot_kws={
        "fontsize": 9.3
    },

    ax=ax_sensitivity
)


# ==================================================================
# 10. Panel titles
# ==================================================================

ax_overall.set_title(
    "A. Overall SSc-ILD cohort",
    fontsize=12,
    fontweight="bold",
    pad=14
)

ax_sensitivity.set_title(
    "B. Laboratory–PFT interval ≤12 months",
    fontsize=12,
    fontweight="bold",
    pad=14
)


# ==================================================================
# 11. Heatmap axis formatting
# ==================================================================

for ax in [ax_overall, ax_sensitivity]:

    ax.set_xlabel("")
    ax.set_ylabel("")
```

```
    ax.tick_params(
        axis="x",
        rotation=0,
        labelsize=11,
        length=0,
        pad=8
    )

    ax.tick_params(
        axis="y",
        left=False,
        labelleft=False
    )

    for tick_label in ax.get_xticklabels():
        tick_label.set_fontweight("bold")


# ============================================================
# 12. Separate pulmonary function parameter labels
#
# The parameter labels are drawn in their own dedicated
# column, so they cannot overlap the analytical group labels.
# ============================================================

ax_labels.set_xlim(0, 1)
ax_labels.set_ylim(10, 0)
ax_labels.axis("off")

for row_index, parameter in enumerate(parameters):

    ax_labels.text(
        0.98,
        row_index + 0.5,
        parameter,
```

```
        ha="right",
        va="center",

        fontsize=9.5,
        color="#252525"
    )


# ================================================================
# 13. Analytical group brackets and group headings
#
# Four groups are shown:
#
#   Row 0      : Last available DLCO measurement
#   Rows 1-3   : Follow-up PFT
#   Rows 4-6   : First-to-follow-up change
#   Rows 7-9   : Annualized change
#
# Group labels are horizontal and wrapped where needed.
# This avoids long vertical labels overlapping adjacent
# groups or pulmonary function parameter names.
# ================================================================

ax_groups.set_xlim(0, 1)
ax_groups.set_ylim(10, 0)
ax_groups.axis("off")


def add_group_bracket(
    ax,
    y_start,
    y_end,
    label,
    color="#006D8F"
):
    """
```

```
Draw a bracket and centered group label.

Coordinates are expressed in the shared heatmap
row system, with 0 at the top and 10 at the bottom.
"""

x_bracket = 0.94
x_cap = 0.85

# Slightly inset the bracket ends
y_top = y_start + 0.10
y_bottom = y_end - 0.10

# Vertical bracket
ax.plot(
    [x_bracket, x_bracket],
    [y_top, y_bottom],
    color=color,
    linewidth=1.7,
    solid_capstyle="round"
)

# Upper cap
ax.plot(
    [x_cap, x_bracket],
    [y_top, y_top],
    color=color,
    linewidth=1.7
)

# Lower cap
ax.plot(
    [x_cap, x_bracket],
    [y_bottom, y_bottom],
    color=color,
    linewidth=1.7
```

```
    )

    # Group heading
    ax.text(
        0.78,
        (y_start + y_end) / 2,
        label,

        ha="right",
        va="center",

        fontsize=9,
        fontweight="medium",
        linespacing=1.15,

        color="#173B49"
    )


add_group_bracket(
    ax_groups,
    0,
    1,
    "Last available\nDLCO measurement"
)

add_group_bracket(
    ax_groups,
    1,
    4,
    "Follow-up PFT"
)

add_group_bracket(
    ax_groups,
    4,
```

```
    7,
    "First-to-follow-up\nchange"
)

add_group_bracket(
    ax_groups,
    7,
    10,
    "Annualized\nchange"
)


# ==============================================================
# 14. Visible separators between analytical groups
#
# Three BLACK horizontal separator lines are drawn:
#
#   y=1 : after DLCO
#   y=4 : after follow-up PFT
#   y=7 : after first-to-follow-up change
# ==============================================================

group_boundaries = [1, 4, 7]

for ax in [ax_overall, ax_sensitivity]:

    for boundary in group_boundaries:

        ax.axhline(
            y=boundary,
            color="#222222",
            linewidth=1.5,
            zorder=10
        )
```

```
# Subtle matching dividers in the parameter label column

for boundary in group_boundaries:

    ax_labels.axhline(
        y=boundary,
        color="#D0D0D0",
        linewidth=0.8
    )


# ============================================================
# 15. Colorbar formatting
# ============================================================

ax_colorbar.set_ylabel(
    "Correlation coefficient (ρ)",
    fontsize=11,
    labelpad=12
)

ax_colorbar.tick_params(
    labelsize=10,
    length=4
)


# ============================================================
# 16. Save publication-quality figure
# ============================================================

output_name = "Figure_2_Heatmap_FIB4_APRI_PFT"

# PNG: high-resolution image
fig.savefig(
    f"{output_name}.png",
```

```
    dpi=600,
    bbox_inches="tight",
    facecolor="white"
)

# PDF: vector format
fig.savefig(
    f"{output_name}.pdf",
    bbox_inches="tight",
    facecolor="white"
)

# TIFF: high-resolution, LZW compression
fig.savefig(
    f"{output_name}.tiff",
    dpi=600,
    bbox_inches="tight",
    facecolor="white",
    pil_kwargs={
        "compression": "tiff_lzw"
    }
)

print("Figure files saved successfully:")
print(f"  {output_name}.png")
print(f"  {output_name}.pdf")
print(f"  {output_name}.tiff")


# ============================================================
# 17. Display figure
# ============================================================

plt.show()
```
